# A highly efficient synthetic strategy for *de novo* NP encapsulation into metal–organic frameworks: enabling further modulated control of catalytic properties†

**DOI:** 10.1039/d3sc05179j

**Published:** 2023-11-07

**Authors:** Li Zhou, Yuanyuan An, Jialong Ma, Guoxiu Hao, Zhehui Li, Junchen Chen, Lien-Yang Chou

**Affiliations:** a School of Physical Science and Technology, ShanghaiTech University Shanghai 201210 China zhuoly@shanghaitech.edu.cn; b Department of Chemistry Merkert Chemistry, Boston College, Chestnut Hill Massachusetts 02467 USA

## Abstract

*De novo* encapsulation is a prevalent method to prepare composite materials where the structure-tunable metal nanoparticles (NPs) are holistically coated with metal–organic frameworks (MOFs). This method has been demonstrated to have promise in various fields but the extensive application of this approach is still challenging. This study proposed, for the first time, leveraging a specific surface-energy-dominated (SED) mechanism to achieve a highly efficient synthetic strategy for *de novo* NP encapsulation. The generality of this strategy is proved in applying to various MOFs, reaction conditions and the use of capping agents. By applying the strategy, Pd NPs with different morphologies are encapsulated in UiO-67, which is prone to self-assembly without coating, and an interesting enhancement is investigated in the selective semihydrogenation of alkynes on different Pd surfaces. These results demonstrate that the control of surface energy is a feasible method for efficient NP encapsulation which sheds light on the rational design of MOF-based composites for future applications.

## Abstract 2

The surface-energy-dominated synthetic strategy to enhance efficiency of NP encapsulation into MOFs.

## Graphical abstract

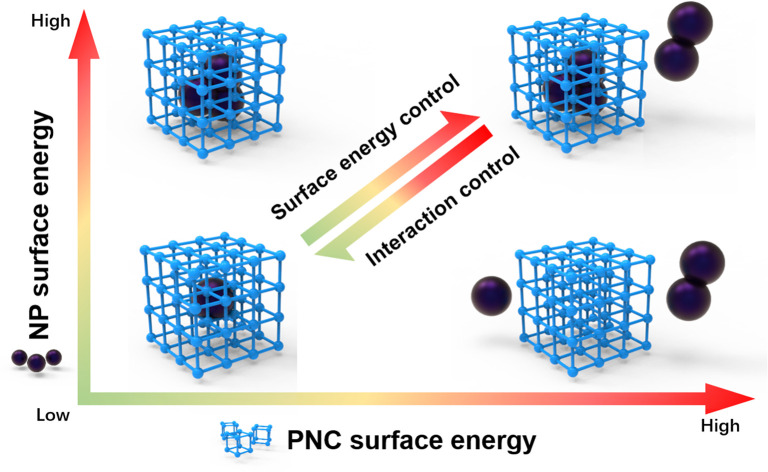

## Introduction

Supporting metal nanoparticles (NPs) by using porous materials is regarded as an effective route to physically isolate them from aggregation owing to their high surface energy, thereby improving their stability.^1^ Metal–organic frameworks (MOFs) with high tunability, a wide variety, and an adjustable pore environment have emerged as a promising porous crystalline material for the encapsulation of NPs, which can provide complementary properties to embedded NPs, *e.g.*, catalytic selectivity,^2^ biocompatibility,^3,4^ and detection sensitivity,^5^ in addition to stability. Besides, MOFs can potentially enhance the performance of the embedded NPs *via* confinement effects^6,7^ and synergy.^8,9^ Therefore, NPs@MOF composites have been widely applied in catalysis,^2^ biology,^3^ medicine,^10^ detection,^11^ carbon neutrality,^9^ environmental protection,^12^*etc.*

Several strategies for the encapsulation of metal NPs into MOFs have been developed.^13–15^ A neat method, referred to as the *de novo* or bottle around ship approach, to encapsulate pre-synthesized NPs into MOFs, has attracted increasing research attention recently.^16–18^ Compared to the common impregnation method, this method intrinsically prevents drawbacks of NP formation on the MOF external surface and the structural damage of MOFs arising from impregnation reduction.^16–18^ Moreover, the *de novo* method not only exhibits the advantage of incorporating metal NPs with controllable size,^19^ morphology^20^ and composition,^21^ but also enables the construction of fully confined NPs with well-defined pore structures of MOFs, including the molecular sieve properties^22–24^ and NP/MOF charge transfer effects.^25–29^

Despite the advantages, extensive applications of the *de novo* method for NP encapsulation are still challenging mostly due to two reasons. First, the conditions for successful NP encapsulation depend on trial and error. It is difficult to find appropriate *de novo* conditions due to the systematic inconsistency in the “synthetic myth”. For example, some studies reported that harsh MOF growth conditions such as high temperatures or a low pH can cause NP self-aggregation.^30^ Second, it is difficult to prevent the formation of pure self-assembled MOF crystals and unembedded NPs during encapsulation, leading to yield reduction.^31–33^ Therefore, only limited types of MOFs are reported for use through the *de novo* method, such as the most common ZIF-8,^19,34^ UiO-66,^35,36^ MIL-53,^37^ and IRMOF-3.^38^ It is of high demand to address the generality of the *de novo* approach, so that more MOF materials, as shells, can be tuned to interact with guest materials, which could expand the inventory of further functional core–shell composite materials.

In this study, we looked at the encapsulation process from a different perspective. Instead of the traditional mediating function, we leveraged the different surface energies in a heterogenous self-assembly and proposed a surface-energy-dominated (SED) synthetic strategy to realize the generality of the *de novo* approach. Inspired by a non-classical crystallization by particle attachment (CPA) mechanism,^39^ which has been demonstrated to be applicable in both metal NP^40,41^ and MOF^42^ synthesis, the *de novo* process can be considered a heterogeneous self-assembly of the metal NPs and MOF-primary nanocrystals (PNCs). The CPA process involves the oriented attachment of pre-formed particles to form the final crystals,^42^ where the surface energy of pre-formed particles is the basic driving force.^43,44^ Therefore, we propose that the surface energy difference between NPs and MOF-PNCs can significantly affect the encapsulation results. The SED synthetic strategy is adjusting the surface energy of metal NPs or MOF-PNCs by using the capping agents to control the competition between heterogeneous and homogeneous self-assembly in the *de novo* process. In addition to the proposed mechanism, the traditional mediating function of the capping agent as a bridge between metal NPs and the MOF^10,19,20,30,45^ was also observed when the interaction effect outperforms the surface-energy effect. The combination of the two encapsulation mechanisms made it possible to fine tune the synthesis conditions allowing for the broad applications of NP/MOF composites by the *de novo* synthesis. As a proof of concept, a MOF-assisted shape-dependent NP catalysis system was established for alkyne selective hydrogenation, which is an important reaction in the chemical industry.^46^ The reaction is greatly influenced by NP surface morphologies^47–49^ and support effects.^50,51^ By applying the SED synthetic strategy, the crystal-facet effect of metal NPs for catalytic performance can be further improved by using MOF supports. The results reveal the applicability of this strategy and the potential of MOFs in regulating metal NPs with different morphologies.

## Results and discussion

**Fig. 1:**
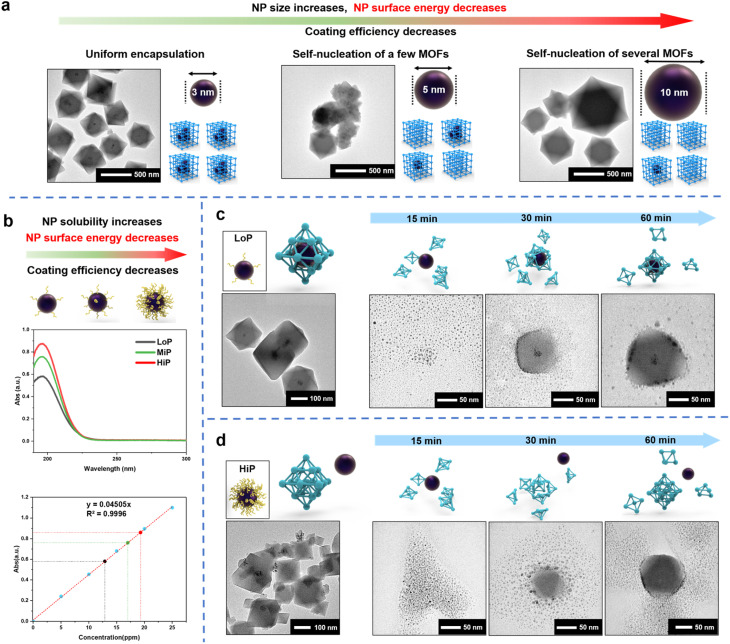
(a) With the increase in the size of Pt NPs, the coating efficiency decreases. (b) UV absorption of PVP on 3 nm LoP (black), 3 nm MiP (green) and 3 nm HiP (red). (c) TEM images of LoP@UiO-66 and time-dependent studies of the synthesis of LoP@UiO-66. (d) TEM images of HiP/UiO-66 and time-dependent studies of the synthesis stages of HiP/UiO-66. A high concentration of capping agents on Pt NPs leads to a decrease in the surface energy.

Polyvinylpyrrolidone (PVP) containing hydrophobic carbon chains and hydrophilic amide groups are not only used to synthesize metal NPs but also act as an effective mediator in the *de novo* approach.^16^ PVP works as an effective MOF coating mediator by strengthening the interaction between NPs and MOF precursors through its amide group which has high binding affinity. Therefore, a common practice is to cover the NP surface with a high concentration of PVP where the MOF coating mediator plays a dominant role in the process. However, the complex MOF coating results suggest that other factors control the process. To demonstrate the complexity of NP encapsulation, different sizes of Pt NPs (3 nm, 5 nm and 10 nm) were synthesized with PVP^52^ (Fig. S1†) and encapsulated into UiO-66 according to a *de novo* method reported previously^36^ (Fig. 1a). Interesting results were observed from the transmission electron microscopy (TEM) images. Pt NPs of 3 nm were uniformly encapsulated into UiO-66, but the encapsulation efficiency decreased with the increase in the size of Pt NPs. A few pristine self-assembled UiO-66 crystals (unloaded pure MOFs) were observed by the addition of 5 nm Pt NPs, and nearly half-unpacked UiO-66 crystals were observed by the addition of 10 nm Pt NPs. This phenomenon revealed that the mechanism of the *de novo* approach is more complex than previously expected and might be related to the surface energy of NPs.

Besides the function of the mediator, PVP can stabilize NPs *via* the reduction of their surface energy by adsorbing on the surface of nanoparticles to change the surface physicochemical properties and improve the stability.^53^ If the MOF coating process is mainly controlled by the surface energy of NPs, less PVP will be more favorable for the coating. In conventional synthesis of NPs, the amount of PVP added is often either overlooked or less calculated. Instead, to support the hypothesis, Pt NPs (3 nm) with different amounts of surface-bound PVP were synthesized. According to the amount of PVP used for NP synthesis, the amount in the reference was labeled as MiP (moderate amount of PVP),^36^ and a low amount and a high amount of PVP were referred to as LoP (Fig. S2a†) and HiP (Fig. S2b†), respectively. Pt NPs with low or high dosage of surface-PVP exhibited different aggregation abilities after centrifugation (Fig. S2a and b†). The high dosage of PVP increased the NP solubility, indicating that interparticle interaction is regulated by the PVP amount. Compared with a low PVP amount on the surface of Pt NPs, a high amount on the particles' surface would further reduce surface energy and increase the stability of particles, leading to highly soluble NPs. UV absorption analysis can be employed to quantitatively confirm the surface-bound PVP on LoP and HiP. The carbonyl absorption peak of PVP at 196 nm linearly represented the PVP concentration of the aqueous solution (Fig. S3a and b†). The amount of PVP on LoP and HiP is defined from the mass ratios of PVP and Pt NPs, corresponding to 34 eq. and 52 eq. respectively, according to UV absorption (Fig. 1b) and elemental analysis (ICP-AES, Table S1†); this result indicated that the content of PVP on the surface of NPs is related to surface energy and can be controlled by the amount of PVP added.

Then, the Pt NPs with different surface-bound PVP contents were used for the UiO-66 encapsulation test. LoP can be well encapsulated in UiO-66 (Fig. 1c). In contrast, a large number of Pt NPs were uncoated and adsorbed on the outside of UiO-66 by using HiP (Fig. 1d). These observations revealed two facts. First, the encapsulation results are strongly affected by the amount of surface-bound PVP. Second, it is easier to encapsulate Pt NPs with less surface-bound PVP than those with more surface-bound PVP, indicating that the coating process is dominated by the surface energy of Pt NPs and that the lower the amount of PVP bound on the surface, the more favorable it is for the MOF coating. Time-dependent studies were conducted to gain insights into the encapsulation process. TEM images of LoP@UiO-66 at different synthesis stages revealed that small UiO-66 PNCs are first be formed in the solution and attached to Pt NPs, followed by aggregation with Pt NPs and then MOF growth (Fig. 1c). The encapsulation process is similar to the CPA process. Therefore, the observation is best explained by the surface energy theory. The high surface energy of Pt NPs can facilitate the hetero-assembly of UiO-66 PNCs. As for HiP/UiO-66 (Fig. 1d), after the formation of small UiO-66 PNCs in the solution, the PNCs were rapidly attached to each other, resulting in the growth of the UiO-66 crystals and subsequent accumulation and adsorption of the Pt NPs on the UiO-66 surface. The results reveal that the attachment of MOF-PNCs is critical for NP encapsulation and that the surface energy of NPs controls encapsulation, which can be observed in the LoP@UiO-66 and HiP/UiO-66 experiments.

From the results of the time-dependent investigation, NPs with a high surface energy (LoP) tended to interact with MOF-PNCs to reduce their surface energy, but the self-assembly of MOF-PNCs to form pristine MOFs was also a possible pathway to reduce the surface energy if the NP surface energy is not high enough (HiP). The discrepancy in the surface energy between metal NPs and MOF-PNC possibly led to different coating results. Therefore, it is hypothesized that in the SED encapsulation mechanism of NPs into MOFs. the MOF-PNCs heterogeneously self-assembled with NPs with a surface energy greater than that of MOF-PNCs (Fig. S4a†). On the other hand, the homogeneous self-assembly of MOF-PNCs was preferred when the surface energy of MOF-PNCs was greater than that of NPs (Fig. S4b†). The SED assumption may provide insight into resolving the typical difficulty in the prevention of unencapsulated MOF crystals *via* the *de novo* approach. For example, if the encapsulation fails, we can potentially change the reaction condition by adjusting the surface energy of NPs to match with the energy needed for driving the encapsulation.

Taking HiP as an example, the surface energy of HiP NPs was less than that of UiO-66 PNCs; hence, it cannot be encapsulated into UiO-66. If we tried to increase the surface energy of NPs, implying to reduce surface-bound PVP content of NPs, a sufficiently high surface energy of NPs could be acquired to achieve successful UiO-66 encapsulation, as demonstrated in the case of LoP@UiO-66. However, this straightforward strategy may not be applicable to the synthetic conditions for all metal NPs due to the tradeoff. First, some NPs require a certain amount of a capping agent to assure uniform growth for narrow size distribution, especially for morphologically controlled metal NPs. Second, NP aggregation due to high surface energy is a common issue when the capping agent is not enough. Hence, there is an intricate window where the amount of capping agent is fine tuned to increase the surface energy of NPs just enough to achieve desirable encapsulation. With that said, in some extreme cases, the capping agent alone is not able to solve the energy mismatch and NP aggregation due to the inherent nature of the NPs and MOF-PNCs.

**Fig. 2:**
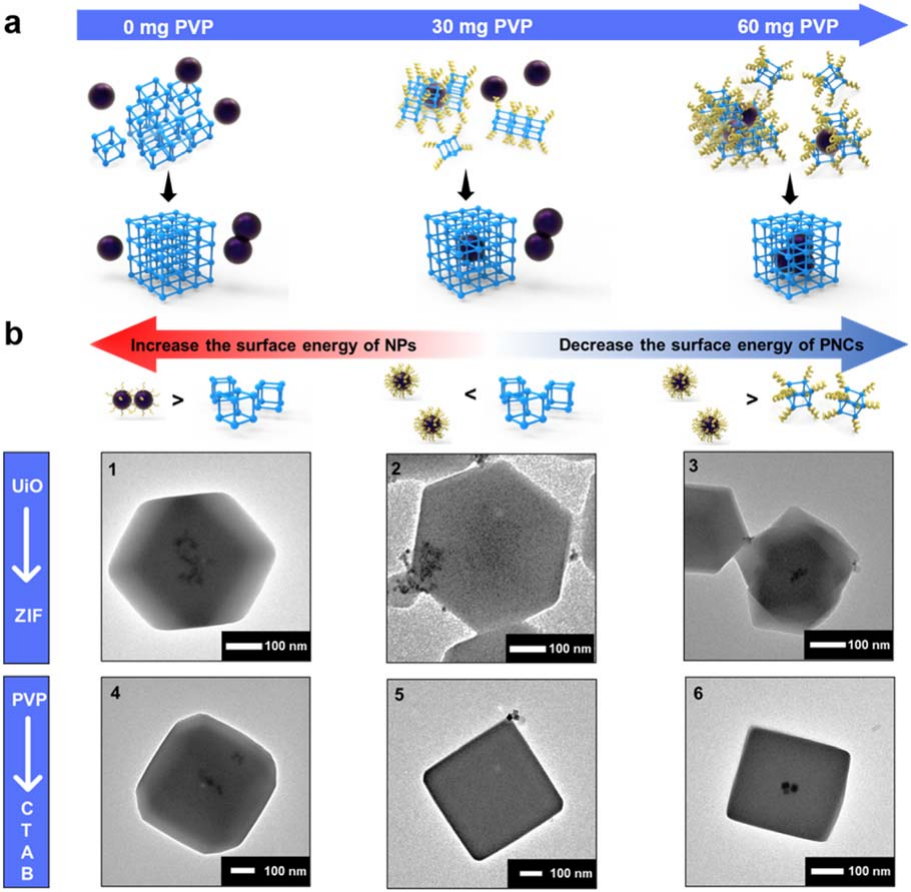
(a) Schematic of the addition of PVP preferentially attaching to UiO-66-PNCs and affecting the results of the heterogeneous self-assembly. (b) Applications of the synthetic strategy to different (1–3) MOFs and (4–6) capping agents. (1) 3 nm LoP@ZIF-8 and (2) 10 nm HiP/ZIF-8 without the addition of free PVP and (3) with the addition of 100 mg of free PVP. (4) 20 nm LoC@ZIF-8 with 0.05 mM CTAB in an aqueous solvent. (5) 20 nm HiC@ZIF-8 with 0.05 mM and (6) with 0.2 mM CTAB in an aqueous solvent.

To find an alternative approach, we start looking into decreasing the surface energy of UiO-66-PNCs to match with that of NPs. By leveraging the NP synthesis strategy and considering the observed phenomenon that adding more capping agents during MOF growth stabilizes smaller MOF crystals,^54,55^ a potential route to reduce the surface energy of MOF-PNCs is to incorporate a capping agent during the MOF synthesis process (Fig. S5†). Here, PVP was selected for convenience and to keep the synthesis conditions simple. The PVP additive was thought to preferentially adhere to the particles, NPs or PNCs, which contained a relatively high surface energy. The results revealed that the heterogeneous self-assembly of HiP and UiO-66-PNCs gradually increases with the increase in the addition of PVP additives during encapsulation (Fig. S6†). As expected, suspended PVP preferentially attached to UiO-66-PNCs due to their higher surface energy than that of HiP NPs; therefore, the PVP additive helps to encapsulate HiP into UiO-66 (Fig. 2a). Notably, excess free PVP additive led to the failure of encapsulation (Fig. S7a†). Excess PVP was speculated to stabilize both PNCs and metal NPs to minimize their surface energy. The main driving force for oriented attachment with excess PVP may now be dominated by the bridging effect of PVP. However, the excess free PVP in solution possibly also attached to MOF-PNCs competitively with NPs, leading to encapsulation failure (Fig. S7b†). This result is consistent with the observation of Huo *et al.*^19^ in that the affinity between NPs and MOFs can be inhibited by free capping agents.

With this result, the applicability of the synthetic strategy was investigated next. Based on our SED hypothesis, the surface energy of UiO-67-PNCs was likely much larger than that of UiO-66-PNCs; hence, it is extremely difficult for NP encapsulation. To test this assumption, the surface-energy difference between UiO-66 and UiO-67 was calculated by density functional theory (DFT) (Fig. S8a†). Bond-breaking enthalpy is used to represent surface energy as surface energy reflects the destruction of intermolecular chemical bonds during the formation of material surfaces.^56,57^ The MOF periodic structure is mainly connected by ligands and coordination bonds of the second building unit (SBU). Therefore, the bond-breaking enthalpy of different ligands and the SBU could reflect the surface-energy level between different MOFs. Both UiO-66 and UiO-67 have fcu topology and are generally stable as octahedral crystals, which means that the {111} facets are exposed.^58^ Nine ligands need to dissociate from the SBU to create a (111) crystal face in a single unit cell (Fig. S8b†). The difference in bond-breaking enthalpy between UiO-67 and UiO-66 is about 4.5 eV. Therefore, the surface energy of UiO-67-PNCs is much greater than that of UiO-66-PNCs. Increasing the surface energy of NPs or decreasing the surface energy of UiO-67-PNCs is expected to overcome the challenge of using UiO-67 in *de novo* encapsulation.

LoP and HiP were used again for the UiO-67 encapsulation test. Even LoP was not encapsulated into UiO-67 (Fig. S9a†), let alone HiP (Fig. S10†). Time-dependent studies of LoP/UiO-67 (Fig. S9b†) revealed phenomena extremely similar to those of HiP/UiO-66 (Fig. S4b†), indicating that the surface energy of UiO-67-PNCs is greater than the energy of LoP. Inspired by HiP@UiO-66, Pt NPs were successfully encapsulated into UiO-67 by the appropriate addition of a PVP additive (Fig. S9c†). Time-dependent studies of LoP@UiO-67 (Fig. S9d†) also revealed a process extremely similar to that of LoP@UiO-66 (Fig. 1c), indicating that the surface energy of UiO-67-PNCs is less than the energy of LoP *via* the help of the PVP additive. Powder X-ray diffraction (PXRD) was conducted and nitrogen sorption isotherms were obtained to demonstrate that the crystallinity and porosity of LoP@UiO-67 are as good as pure UiO-67 crystals (Fig. S11a and b†). Elemental mapping images from energy-dispersive X-ray spectroscopy (EDS) revealed that Pt NPs are completely confined inside UiO-67 (Fig. S11c†).

To extend the scope of the synthetic strategy, ZIF-8 was selected, corresponding to other popular MOFs, but extremely different from the UiO series in terms of the node, ligands, and topology.^59^ In addition, the synthesis conditions of ZIF-8, which can be synthesized in an aqueous solution at room temperature, were considerably much milder than those of UiO-66. The effects of synthetic conditions such as solvent, pH, and temperature were therefore excluded. The results revealed that Pt NPs, whether LoP or HiP, can be well encapsulated into ZIF-8 (Fig. 2b1 and S12a†), indicating that the surface energy of ZIF-8 is less than that of UiO-66. To investigate our theory, 10 nm HiP Pt NPs (Fig. S12b and Table S1†) were synthesized to further reduce the surface energy of NPs. Owing to their low surface energy, 10 nm HiP was not encapsulated into ZIF-8, but with the help of free PVP, it was successfully re-encapsulated into ZIF-8 (Fig. 2b2 and b3). The results indicated that the SED mechanism is universal and is not affected by MOFs with completely different topologies and different synthetic conditions.

The results encouraged us to further explore whether NPs synthesized using different capping agents followed a similar mechanism. By using cetyltrimethylammonium bromide (CTAB) as the capping agent, 20 nm Pd NPs with different concentrations of CTAB, named LoC and HiC (Fig. S13a and b†), were synthesized. UV absorption analysis can also be employed to quantitatively confirm the surface-bound CTAB on LoC and HiC, and the amount of CTAB on LoC and HiC is defined from the mass ratios of CTAB and Pt NPs, corresponding to 1 eq. and 2.4 eq. respectively, according to UV absorption (Fig. S14†) and elemental analysis (ICP-AES, Table S1†). LoC was found to be well encapsulated into ZIF-8 (Fig. 2b4). Although HiC cannot be encapsulated due to its low surface energy, as expected, the addition of an appropriate amount of free CTAB in the synthesis can reduce the surface energy of ZIF-8-PNCs to achieve successful encapsulation (Fig. 2b5 and 3b6). The encapsulation process can be regulated by using the amount of surface bound CTAB, and the additional free CTAB can decrease the surface energy of MOF-PNCs to improve the coating efficiency of HiC, all of which are consistent with the behavior exhibited by PVP. The results suggested that the surface energy-dominated mechanism is also applicable to various capping agents.

**Fig. 3:**
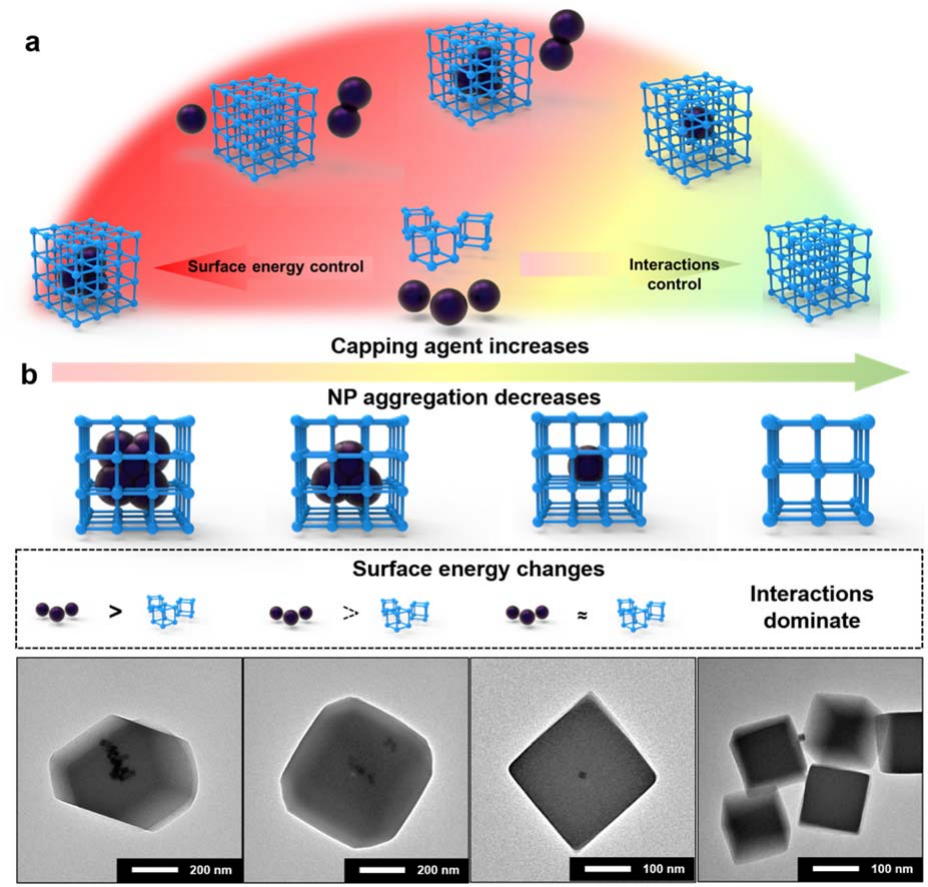
(a) Summary of the mechanism of *de novo* NP encapsulation. (b) When the surface energy of metal NPs is greater than that of MOF-PNC, the addition of free end capping agents leads to a specific core–shell structure due to the fine-tuning of the surface energy difference.

Based on the results obtained above, the mechanism of NP encapsulation is summarized (Fig. 3a and S15†). Generally, heterogeneous self-assembly occurs when the surface energy of metal NPs is greater than that of MOF-PNCs; otherwise, homogeneous self-assembly occurs (Fig. S15a and b†). In certain cases, the high surface energies of NPs and MOF-PNCs result in the heterogeneous and homogeneous self-assembly simultaneously, in other words, incomplete NP encapsulation (Fig. S15c†). When the surface energies of NPs and MOF-PNCs were low, the interaction of the capping agents and MOF-PNCs became dominant (Fig. S15d†). Therefore, to successfully realize *de novo* encapsulation, a low dosage of capping agents is preferred in NP synthesis conditions, or the excess capping agents covering the NPs should be washed off extensively after NP generation. In some cases, the low surface energy of metal NPs, or the high surface energy of MOF-PNCs, led to MOF self-assembly without encapsulating NPs. To solve this problem, additional capping agents can be added to the system to reduce the surface energy of PNCs and finally achieve NP encapsulation. With the SED synthetic strategy, MOF materials that are otherwise limited in the *de novo* approach may be applied. Besides UiO-67, HKUST-1 and MIL-101(Fe) were considered to be difficult for NP encapsulation, possibly due to the acidic environment generated by ligand dissociation^32^ or the instability of Pt colloids during a long–term reaction at high temperatures.^60^ However, in our cases, both of them achieved Pt@HKUST-1 and Pt@MIL-101(Fe) under the guidance of the SED encapsulation mechanism (Fig. S16†). Owing to their high surface energy, 3 nm LoP Pt NPs were encapsulated into HKUST-1 and MIL-101 without any other operation (Fig. S16a and d†). To verify the effect of the surface energy on the two MOFs, 10 nm HiP Pt NPs were investigated. The uncoated and MOF self-assembly phenomena were observed (Fig. S16b and e†). Pt@HKUST-1 and Pt@MIL-101(Fe) were achieved again with 10 nm HiP Pt after the addition of an appropriate amount of PVP to reduce the surface energies of MOF-PNCs (Fig. S16c and f†). Therefore, the universal surface-energy dominated synthetic strategy is demonstrated.

By the fine tuning of the surface energy difference between NPs and MOFs, some special structures of NP@MOF composites can be further prepared. Taking LoP@UiO-66 as an example, if free PVP was added under the encapsulation conditions, the distribution of Pt NPs in MOFs gradually dispersed with the increase in the PVP addition (Fig. S17†). The results revealed that PVP selectively attaches to particles with a relatively high surface energy, corresponding to Pt NPs here, which is in agreement with the SED mechanism. With the decrease in the surface energy of Pt NPs, the ability of heterogeneous self-assembly decreases, leading to their increased dispersion in MOFs. Similar results were observed in the case of LoC@ZIF-8, where with the increase in CTAB addition, the dispersity of Pd NPs increased, even resulting in one-to-one encapsulation (Fig. 3b). This result was possibly attributed to the fact that the surface energy of LoC is just slightly greater than that of ZIF-8 under this condition. Meanwhile, the result may also be attributed to the capping agents acting as heterogeneous nucleation sites to allow *in situ* growth of MOFs.^61^ This means that when the surface energy difference cannot be the main driving force, interactions between the capping agent and MOF would dominate the encapsulation. Moreover, the addition of excess capping agents resulted in no coating of the NPs by MOFs (Fig. 3b and S17†). The results are also consistent with previous results obtained for the encapsulation of HiP with free PVP in that excess capping agents can inhibit the interaction-dominated self-assembly.

To demonstrate the advantage of the encapsulation method, a MOF-assisted shape-dependent NP catalysis system was established for alkyne selective hydrogenation. The semihydrogenation of alkynes into alkenes is of great importance in pharmaceuticals, vitamins, agrochemicals, fragrances, polymerization, olefin metathesis reactions, and so on.^46^ Pd-based catalysts have been recognized as one of the most effective catalysts for the semihydrogenation of alkynes due to their high activity and low operating temperature, but Pd still faces significant challenges in overhydrogenation.^62^ Recent studies have found that the catalytic performance of selective hydrogenation of alkynes is greatly influenced by the adsorption/desorption strength of reactants, intermediates and products on the surface of palladium nanoparticles, which can be regulated by Pd surface morphologies^47–49^ and support effects.^50,51^ MOF-based Pd catalysts, especially UiO series have been proven to be good supports for Pd NPs in improving the selective hydrogenation of alkynes due to the electron transfer from the Pd NPs to the electron-withdrawing MOF nodes.^50^ In this regard, the combination of Pd morphology and the MOF support effect may further optimize the catalytic performance of selective hydrogenation of alkynes. However, to the best of our knowledge, there is a lack of scientific studies on achieving MOF embedding metal NPs with different crystal shapes in current encapsulation methods.

**Fig. 4:**
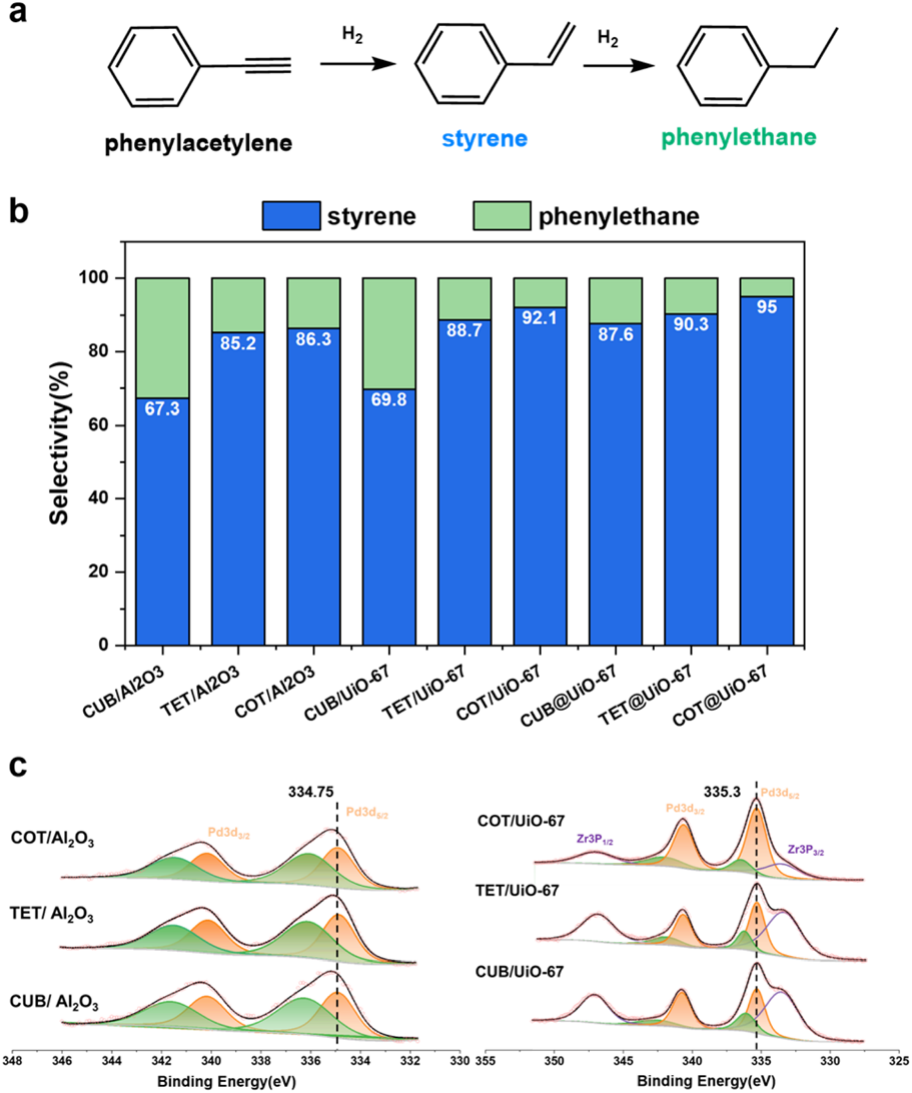
(a) Selective hydrogenation of PhA. (b) St selectivity of cubic, tetrahedral and cuboctahedral Pd NPs on the alumina support, on the surface of UiO-67 and coated with UiO-67. (c) XPS data of CUB/Al_2_O_3_, TET/Al_2_O_3_, and COT/Al_2_O_3_, and CUB/UiO-67, TET/UiO-67, and COT/UiO-67.

The cubic, tetrahedral and cuboctahedral Pd NPs are synthesized (Fig. S18a–c and Table S1†) and successfully encapsulated into UiO-67, labeled as CUB@UiO-67, TET@UiO-67 and COT@UiO-67, respectively. (Fig. S18d–I and Table S2†) For comparison, the cubic, tetrahedral and orbicular Pd NPs, respectively, are loaded on the surface of alumina or UiO-67, labeled as CUB/Al_2_O_3_, TET/Al_2_O_3_, and COT/Al_2_O_3_ (Fig. S18j–l†) or CUB/UiO-67, TET/UiO-67, and COT/UiO-67. (Fig. S18m–o†) The selective hydrogenation of phenylacetylene (PhA) to styrene (St) is used to evaluate the catalytic performance of the composite catalysts (Fig. 4a). The conversion rate is controlled to be above 90% as the criterion for evaluating catalytic selectivity due to the competitive adsorption of PhA and St at a low conversion rate, which may lead to an increase in semi-hydrogenation selectivity (Fig. S19†). We first compare catalytic performances of different morphological Pd NPs on an alumina support. The cubic Pd nanoparticles show the lowest St selectivity, only 67.3%. On the other hand, both the tetrahedral and cuboctahedral Pd nanoparticles exhibit similar but higher St selectivity than the cubic Pd nanoparticles, of 85.2% and 86.3%, respectively (Fig. 4b). These results suggest that the cubic Pd NPs have a strong adsorption of St due to more exposed {100} facets, making them prone to overhydrogenation and resulting in lower selectivity. In contrast, the tetrahedral and cuboctahedral nanoparticles mainly expose {111} facets, leading to relatively weak adsorption of St and generation of more St products. The observations are consistent with the reported calculation results.^63^ When using Pd NPs supported on the surface of UiO-67 for the same PhA hydrogenation reaction, selectivity differences still exist between the crystal facets of Pd NPs. The trend of St selectivity of cubic, tetrahedral and cuboctahedral Pd NPs on the surface of UiO-67 is the same as that of an alumina support, but they are all increased, 69.8%,88.7% and 92.1%, respectively (Fig. 4b). XPS data show that the binding energy of the 3d_5/2_ orbital of Pd NPs loaded on the surface of UiO-67 is 335.3 eV, which is higher than the binding energy of that loaded on the surface of alumina, 334.75 eV (Fig. 4c), indicating that the Pd NPs loaded on UiO-67 are in an electron deficient state. Pd NPs in more electron-deficient states have been proved to have higher St selectivity.^50^ Then we used the Pd NPs encapsulated in UiO-67 for the same reactions and some interesting results were observed. Firstly, Pd NPs coated with UiO-67 further enhance the selectivity of St on each crystal facet. St selectivity of the cubic, tetrahedral, and cuboctahedral Pd NPs are all increased to 87.6%, 90.3%, and 95%, respectively (Fig. 4b). We speculate that the more the number of contact sites between Pd nanoparticles and electron-withdrawing nodes of UiO-67, the higher the enhancement of UiO-67 for St selectivity of Pd NPs. Therefore, complete MOF coating enables more efficient electron transfer from Pd NPs to UiO-67 than that on the MOF surface although the electron transfer information of Pd NPs embedded in a MOF cannot be obtained by XPS due to the thick MOF shell. Secondly, the selectivity of St on cubic Pd nanoparticles is dramatically improved by nearly 20%. Based on the same assumptions of contact sites, the {100} facet has more surface atoms than the other facets so the enhancement of St selectivity is also higher. The results show that the design of MOF catalysts cannot ignore the synergistic differences between the different facets of metal NPs and MOF supports, especially for NP structure sensitive catalytic reactions. Therefore, choosing appropriate MOF supports to regulate the morphology effect of metal NPs will further promote the development of new core–shell MOFs in the catalysis field.

## Conclusions

In summary, we have demonstrated a new strategy for the *de novo* encapsulation of NPs into MOFs. The surface energy of metal NPs or MOF-PNCs was adjusted in this strategy to realize heterogeneous self-assembly. The strategy provided a simple approach to control encapsulation, which was demonstrated to be universal for various MOFs, synthesis conditions, and capping agents. In addition, the synthesis of several MOFs considered to be extremely difficult through the *de novo* approach was achieved by this strategy to prove the generality. Benefiting from the structural and synergistic complexity, the encapsulated Pd nanoparticles with different crystal facets reveal different degrees of impact from their MOF shell. With the development of MOF materials, they have become increasingly important as supporting materials.† Rich and interesting synergies emerge when different MOF hosts interact with various forms of guest species. The contributions of this study are expected to provide insights into the mechanistic investigation of heterogeneous self-assembly and further explore the applications of NPs@MOF composites.

## Data availability

The data that support the findings of this study are available from the corresponding author upon reasonable request.

## Author contributions

L.-Y. C. conceived and developed this study. L. Z. performed the main experiments and analyzed the data. Y. A. provided guidance of the research idea and catalysis experiment. J. M. performed NMR experiments. G. H. performed XPS experiments. Z. L. and J. C. participated in the revision of the article. The manuscript was written by L. Z. and L.-Y. C. with input from all authors.

## Conflicts of interest

There are no conflicts to declare.

## Supplementary Material

SC-014-D3SC05179J-s001
