# Supplementary material for: A highly efficient synthetic strategy for de novo NP encapsulation into metal–organic frameworks: enabling further modulated control of catalytic properties
Source: Chem Sci. 2023 Nov 7;14(45):13126–33. doi: 10.1039/d3sc05179j (PMC10664540; doi:10.1039/d3sc05179j)
Supplement: SC-014-D3SC05179J-s001 [file SC-014-D3SC05179J-s001.pdf]

Electronic Supplementary Material (ESI) for Chemical Science.
This journal is © The Royal Society of Chemistry 2023

Supporting Information

A highly efficient synthetic strategy for *de novo* NP encapsulation into metal-organic frameworks: enabling further modulated control of catalytic properties

Li Zhou,[a] Yuanyuan An,[a] Jialong Ma,[a] Guoxiu Hao,[a] Zhehui Li,[b] Junchen Chen[a] and Lien-Yang Chou[a]*

[a] School of Physical Science and Technology, ShanghaiTech University,Shanghai 201210, China.
[b] Department of Chemistry, Merkert Chemistry, Boston College, Chestnut Hill, Massachusetts, 02467, United States
*E-mail: zhuoly@shanghaitech.edu.cn; orcid.org/0000-0001-8171-1346;

Table of Contents

1. Experimental Section .... Error! Bookmark not defined.

1.1 Materials .... **Error! Bookmark not defined.**

1.2 Synthetic Procedures .... **Error! Bookmark not defined.**

1.3 DFT calculation 8

1.4 Semihydrogenation of alkynes 9

2. Supporting Figures 10

3. Supporting Tables 28

4. References 30

1. Experimental Procedures

1.1. Materials

Ammonium hexachloroplatinate(IV) ($(NH_4)_2PtCl_6$, 99%; Sigma-Aldrich), ammonium tetrachloroplatinate(II) ($(NH_4)_2PtCl_4$, 99%; Sigma-Aldrich), sodium tetrachloropalladate (II) (Na_2PdCl_4, Adamas-beta, 36.4% Pd), polyvinylpyrrolidone (PVP, aladdin, Mw~29000), hexadecyltrimethylammonium bromide (CTAB, Sigma-Aldrich, 99%), ethylene glycol (EG, aladdin, SP, ≥99%), tetramethylammonium bromide (TMABr, TCI, ≥97.0%), Zirconium chloride ($ZrCl_4$, aladdin, 98.0%), iron(III) chloride hexahydrate ($FeCl_3 \cdot 6H_2O$, 97wt%) 1,4-benzenedicarboxylic acid (BDC, aladdin, 99.0%), 4,4′-biphenyldicarboxylic acid (BPDC, aladdin, 98.0%), N, N-dimethylformamide (DMF, GENERAL-REAGENT, >99.5%), acetic acid (GENERAL-REAGENT, >99.5%), zinc nitrate hexahydrate ($Zn(NO_3)_2 \cdot 6H_2O$, Alfa Aesar, 99.6%), 2-methylimidazole (2-mim, Alfa-Aesar, 97%), copper (II) nitrate trihydrate ($Cu(NO_3)_2 \cdot 3H_2O$, 99.5%, Sigma-Aldrich), copper(II) acetate monohydrate ($Cu(OAc)_2 \cdot H_2O$, 99%, Sigma-Aldrich) and 1,3,5-benzenetricarboxylic acid (BTC, 98%, Sigma-Aldrich), methanol (GENERAL-REAGENT, AR), are purchased from the mentioned sources and used without further purification. Ultrapure deionized water (DI water, 18.2 MΩ) used in this study is from Milli-Q Direct 8/16 system. Phenylacetylene (GENERAL-REAGENT, >99.5%), Hydrogen (H_2, 99.999%) are used for heterogeneous catalysis.

1.2. Synthetic procedures

Synthesis of 3 nm, 5 nm, 10 nm Pt-PVP NPs: Pt-PVP NPs were synthesized based on a previous report with some modifications.[1] A total of 0.15 mmol of Pt ions $(NH_4)_2Pt$ (IV)Cl_6 and $(NH_4)_2Pt$ (II)Cl_4), 2.25 mmol of tetramethylammonium bromide, and 3.00 mmol of poly(vinylpyrrolidone) (in terms of the repeating unit; M_w 29 000) were dissolved into 30 mL of ethylene glycol in a 50 mL round-bottom flask at room temperature. The Pt (IV) to total Pt (Pt (II) + Pt (IV)) molar ratios were 0, 20%, and 100% for synthesis of 3 nm, 5 nm, 10 nm NPs, respectively. The mixed solution was heated to 180 °C in an oil bath at 60 °C/min. For 5 nm Pt NPs, the Pt (IV) to Pt (Pt (II) + Pt (IV)) molar ratio was 0.2, and the temperature

was 140 °C. The solutions were held at these respective temperatures for 20 min under argon protection and magnetic stirring, resulting in a dark brown solution. After the solution was cooled to room temperature, acetone (270 mL) was then added to form a cloudy black suspension, which was separated by centrifugation at 5000 rpm for 10 min. The black product was collected by discarding the colorless supernatant. The products were further washed three times by precipitation/dissolution (redispersed in 50 mL of ethanol and then precipitated by adding 200 mL of hexanes). The nanocrystals were then redispersed in 30 mL of DMF for characterization and catalyst preparation.

Synthesis of LoP and HiP Pt-PVP NPs: A total of 0.15 mmol of Pt ions $(NH_4)_2Pt(II)Cl_4$), 2.25 mmol of tetramethylammonium bromide, and 2.00 mmol (2.00 mmol for LoP and 5.00 mmol for HiP) of poly(vinylpyrrolidone) (in terms of the repeating unit; M_w 29 000) were dissolved into 30 mL of ethylene glycol in a 50 mL round-bottom flask at room temperature. The mixed solution was heated to 180 °C in an oil bath at 60 °C/min. The solutions were held at these respective temperatures for 20 min under argon protection and magnetic stirring, resulting in a dark brown solution. After the solution was cooled to room temperature, acetone (270 mL) was then added to form a cloudy black suspension, which was separated by centrifugation at 5000 rpm for 10 min. The black product was collected by discarding the colorless supernatant. The products were further washed three times by precipitation/dissolution (redispersed in 50 mL of ethanol and then precipitated by adding 200 mL of hexanes). The nanocrystals were then redispersed in 30 mL of DMF for characterization and catalyst preparation.

Synthesis of LoC and HiC Pd-CTAB NPs: Pd-CTAB NPs were synthesized based on a previous report with some modifications.[2] 20 mg (20 mg for LoC and 50 mg for HiC) of CTAB was dissolved in 9.5 mL of deionized water (DI water), followed by adding 0.3 mL of 0.01 M H_2PdCl_4 solution. The above solution was placed in 95 °C-oil bath for 5 min. Then, 200 µL of 0.04 M ascorbic acid was added with stirring at 200 rpm. The reaction mixture was stirred for 30 min at 95 °C. After cooling to room temperature, the as-synthesized Pd-CTAB NPs were washed once with DI water via centrifugation (8000 rpm, 10 min). Normally, 10 batches of Pd-CTAB NPs were synthesized and dispersed in 5.0 mL of DI water, yielding a 10 mM Pd-CTAB NPs suspension. For the encapsulation, 0.25 mL of the Pd-CTAB NPs suspension

was centrifuged (8000 rpm, 10min) and dispersed in 0.5 mL DI water.

Synthesis of cubic Pd-PVP NPs: Cubic Pd-PVP NPs were synthesized based on a previous report with some modifications.[3] In a typical synthesis of Pd cubes/bars with different sizes, 8.0 mL of an aqueous solution containing poly(vinyl pyrrolidone) (PVP,MW ≈ 55 000, 480 mg, Aldrich), L-ascorbic acid (AA, 60 mg, Aldrich), and different amounts of KBr and KCl were placed in a 20 mL vial, and pre-heated in air under magnetic stirring at 80 °C for 10 min. Then, 3.0 mL of an aqueous solution containing Na_2PdCl_4 (57 mg, Aldrich) was added using a pipette. After the vial had been capped, the reaction was allowed to proceed at 80 °C for 3 h. The product was collected by centrifugation and washed with acetone and ethanol three times; the products were re-dispersed in 8mL of DMF.

Synthesis of cubobtahedral Pd-PVP NPs: Cubobtahedral Pd-PVP NPs were synthesized based on a previous report with some modifications.[4] The cuboctahedral shape were prepared at 160 ^{o}C in EG. In a standard procedure, 130 mg of PVP and 2 mL of EG were added into a 20 mL glass vial, and heated in an oil bath at 160 ^{o}C under magnetic stirring. Afterwards, 1 mL of EG solution containing 15.5 mg of $Na_2PdCl_4$was quickly injected with a pipette. The vial was removed from the oil bath 3 h later and then cooled down to room temperature. After centrifugation and washing with acetone and ethanol three times, the products were re-dispersed in 3mL of DMF.

Synthesis of tetrahedral Pd-PVP NPs: Tetrahedral Pd-PVP NPs were synthesized based on a previous report with some modifications.[4] The cuboctahedral shape were prepared first as seed. In a standard procedure, 30 mg of PVP and 2 mL of EG were added into a 20 mL glass vial, and heated in an oil bath at 160 ^{o}C under magnetic stirring. Afterwards, 1 mL of EG solution containing 15.5 mg of $Na_2PdCl_4$was quickly injected with a pipette. The vial was removed from the oil bath 3 h later and then cooled down to room temperature. After centrifugation and washing with acetone and ethanol three times, the seeds were re-dispersed in 3mL of EG. Then, 500 mg of PVP, 1 mL of the cuboctahedral Pd seeds (in EG), and 25 mL of TTEG were added into a 100 mL glass vial, and then heated to 140 ^{o}C in an oil bath under magnetic stirring. Meanwhile, 5 mL of TTEG solution containing 20 mg of $Pd(acac)_2$ was prepared for the synthesis of Pd tetrahedrons. After the precursor had been completely dissolved, it was quickly injected into the

vial with a pipette. The reaction was allowed to continue at 140 ^{o}C for 1 h and then cooled down to room temperature. After centrifugation and washing with acetone and water three times, the product was collected and stored in DMF.

Synthesis of cubic, tetrahedral and cubobtahedral Pd-PVP NPs on alumina: 0.005 mmol of cubic, tetrahedral or cubobtahedral Pd-PVP NPs and 40 mg alumina were dispersed in 5 mL ethanol, respectively, and stirring for 3 h.

Synthesis of cubic, tetrahedral and cubobtahedral Pd-PVP NPs on UiO-67: 0.005 mmol of cubic, tetrahedral or cubobtahedral Pd-PVP NPs and 40 mg UiO-67 were dispersed in 5 mL ethanol, respectively, and stirring for 3 h.

Synthesis of UiO-66: UiO-66 were synthesized based on a previous report with some modifications. [5] 41.53 mg of BDC, 58.26 mg of $ZrCl_4$ and 1.37 ml of acetic acid were dissolved in 10 mL of DMF and placed in 120 °C oven for a day. The resulting UiO-66 was washed three times with DMF using a centrifuge (8,000 rpm for 5 min) and sonication and dispersed in 10 mL DMF.

Synthesis of Pt@UiO-66: Pt@UiO-66 were synthesized based on a previous report with some modifications.[5] 41.53 mg of BDC, 58.26 mg of $ZrCl_4$ and 1.37 ml of acetic acid were dissolved in 10 mL of DMF. Then 0.3 ml of Pt in DMF (1 mg mL^{-1}) was added and placed in 120 °C oven for a day. The resulting Pt@UiO-66 was washed three times with DMF using a centrifuge (8,000 rpm for 5 min) and sonication and dispersed in 10 mL DMF.

Synthesis of Pt@UiO-66 with addictive PVP: Pt@UiO-66 were synthesized based on a previous report with some modifications.[5] 41.53 mg of BDC, 50 mg, 100 mg, 200 mg or 500 mg PVP (M_w 29 000), 58.26 mg of $ZrCl_4$ and 1.37 ml of acetic acid were dissolved in 10 mL of DMF. Then 0.3 ml of Pt in DMF (1 mg mL^{-1}) was added and placed in 120 °C oven for a day. The resulting Pt@UiO-66 was washed three times with DMF using a centrifuge (8,000 rpm for 5 min) and sonication and dispersed in 10 mL DMF.

Synthesis of UiO-67: UiO-67 were synthesized based on a previous report with some modifications. [6] 150 mg of BPDC were dissolved in 50 mL of DMF in 120 °C for 10 mins, then cooling to room temperature.100 mg of $ZrCl_4$ and 3 mL of acetic acid were dissolved in the solution above and placed in

120 °C oven for a day. The resulting UiO-67 was washed three times with DMF using a centrifuge (8,000 rpm for 5 min) and sonication and dispersed in 20 mL DMF.

Synthesis of Pt@UiO-67: 150 mg of BPDC and 2 g PVP (M_w 29 000) were dissolved in 50 mL of DMF in 120 °C for 10 mins, then cooling to room temperature.100 mg of $ZrCl_4$ and 3 mL of acetic acid were dissolved in the solution above and Then 3 ml of Pt in DMF (1 mg mL^{-1}) placed in 120 °C oven for a day. The resulting UiO-67 was washed three times with DMF using a centrifuge (8,000 rpm for 5 min) and sonication and dispersed in 20 mL DMF.

Synthesis of Pt@MIL-101(Fe): MIL-101 were synthesized based on a previous report with some modifications.[7] 93.4 mg $FeCl_3{\cdot}6H_2O$, 57.5 mg BDC and 0 mg (for 3 nm LoP) or 100 mg (for 10 nm HiP) PVP (M_w 29 000) were dissolved in 16 mL DMF. Then 0.3 ml of different Pt NPs in DMF (1 mg mL^{-1}) was added and placed in 120 °C oven for a day. After cooling to room temperature, the solid products were collected by centrifugation at 5000 rpm for 10 min and washed twice with DMF. Finally, the obtained MIL-101(Fe) was redispersed in 12 mL DMF for further use.

Synthesis of Pt@HKUST-1: HKUST-1 were synthesized based on a previous report with some modifications.[8] 38.6 mg Cu $(NO_3)_2{\cdot}3H_2O$ and 21 mg 1,3,5-benzenetricarboxylic acid were dissolved in 4.1 mL methanol. Then 0.9 mL Cu $(OAc)_2{\cdot}H_2O$ (0.8 mg/mL) and 0.1 mL different Pt NPs in methanol (1 mg mL^{-1}) were added and placed at 25 °C for 6 hours. the solid products were collected by centrifugation at 5000 rpm for 10 min and washed twice with methanol. Finally, the obtained Pt@HKUST-1 was redispersed in 5 mL methanol for further use.

Synthesis of Pt@ZIF-8: The synthesis was modified from the previous method developed by our group. [9] 113.52 mg of 2-mim was dissolved in 1.75 mL of 0.05 mM, 0.2 mM (one-to-one encapsulation), or 0.5 mM (excessive addition) CTAB aqueous solution to obtain a 2-mim/CTAB solution. 250 µL of 10 mM metal-PVP/CTAB NPs were centrifuged (8000 rpm, 10 min) and dispersed in 500 µL of DI water. To form NP@ZIF-8 with a direct interface, 1.75 mL of 2-mim/CTAB solution was mixed with 0.25 mL of 97.5 mM Zn $(NO_3)_2$ solution in a 20-mL glass vial while stirring at 250 rpm at room temperature. After 10 s, 0.5 mL of metal-CTAB NPs in DI water was added into the above mixture. After stirring for 5 min,

the mixture was left undisturbed for 3 h at room temperature (22.0 ± 1.0 ˚C). The sample was collected by centrifugation (8000 rpm, 10 min). The sample was washed with methanol three times and was dried under a vacuum condition for 24 h.

Synthesis of Pd@ZIF-8: The synthesis was modified from the previous method developed by our group. [9] 113.52 mg of 2-mim was dissolved in 1.75 mL of 0.05 mM, 0.2 mM (one-to-one encapsulation), or 0.5 mM (excessive addition) CTAB aqueous solution to obtain a 2-mim/CTAB solution. 250 µL of 10 mM metal-PVP/CTAB NPs were centrifuged (8000 rpm, 10 min) and dispersed in 500 µL of DI water. To form NP@ZIF-8 with a direct interface, 1.75 mL of 2-mim/CTAB solution was mixed with 0.25 mL of 97.5 mM Zn $(NO_3)_2$ solution in a 20-mL glass vial while stirring at 250 rpm at room temperature. After 10 s, 0.5 mL of metal-CTAB NPs in DI water was added into the above mixture. After stirring for 5 min, the mixture was left undisturbed for 3 h at room temperature (22.0 ± 1.0 ˚C). The sample was collected by centrifugation (8000 rpm, 10 min). The sample was washed with methanol three times and was dried under a vacuum condition for 24 h.

Synthesis of cubic, tetrahedral and cubobtahedral Pd-PVP NPs in UiO-67: 150 mg of BPDC and 2 g PVP (M_w 29 000) were dissolved in 50 mL of DMF in 120 °C for 10 mins, then cooling to room temperature.100 mg of $ZrCl_4$ and 3 mL of acetic acid were dissolved in the solution above and Then 3 ml of cubic, tetrahedral or cubobtahedral Pd-PVP NPs in DMF (1 mg mL^{-1}) placed in 120 °C oven for a day. The resulting UiO-67 was washed three times with DMF and ethanol, then using a centrifuge (8,000 rpm for 5 min) and sonication and dispersed in 20 mL ethanol.

1.3. DFT calculation

First-principles calculations were performed using the CP2K software package with the projector augmented wave method (PAW). A spin-polarized generalized gradient approximation (GGA) was employed to the revised Perdew–Burke–Ernzerhof (PBE) functional for the exchange correlation potentials. We build Zr-SBU, BDC, BPDC, BDC-Zr-SBU, and BPDC-Zr-SBU models respectively. The vacuum layers in z-direction were set to more than 15 Å to ensure the interaction was eliminated. In geometry optimizations, the cut-off energy of the plane wave basis set was 400 eV, all the atomic

coordinates were fully relaxed up to the residual atomic forces smaller than 0.05 eV/Å, and the total energy was converged to 10^{-5} eV. The Monkhorst mesh was 1×1×1. The bond-breaking enthalpy (BBE) is calculated by the formula below.

$$E_{(BBE)} = -[E_{(Ligand\text{-}Zr\text{-}SBU)} - (E_{(Ligand)} + E_{(Zr\text{-}SBU)})]$$

1.4. Semi hydrogenation of alkynes

Semi hydrogenation of alkynes was carried out in ethanol solution under a static hydrogen atmosphere (5 bar). In a typical experiment, 30 μL phenylacetylene was added into 5 mL methanol solution. The catalyst containing 0.098 mg Pd was then added to the mixture. After sonication, the mixture was transferred to a glass vial and placed in the high-pressure laboratory reactor (Julabo). After the reactor was purged with H_2 for 3 times, the reaction was allowed to proceed under stirring at 25 ^{o}C and 5 bar initial H_2 filling pressure. After reacting for various amount of time, the mixture was filtered for NMR analysis. The conversion rate was controlled above 90% as the criterion for evaluating catalytic selectivity due to the competitive adsorption of PhA and St at low conversion rate, which may lead to an increase in semi-hydrogenation selectivity.

2. Supporting Figures

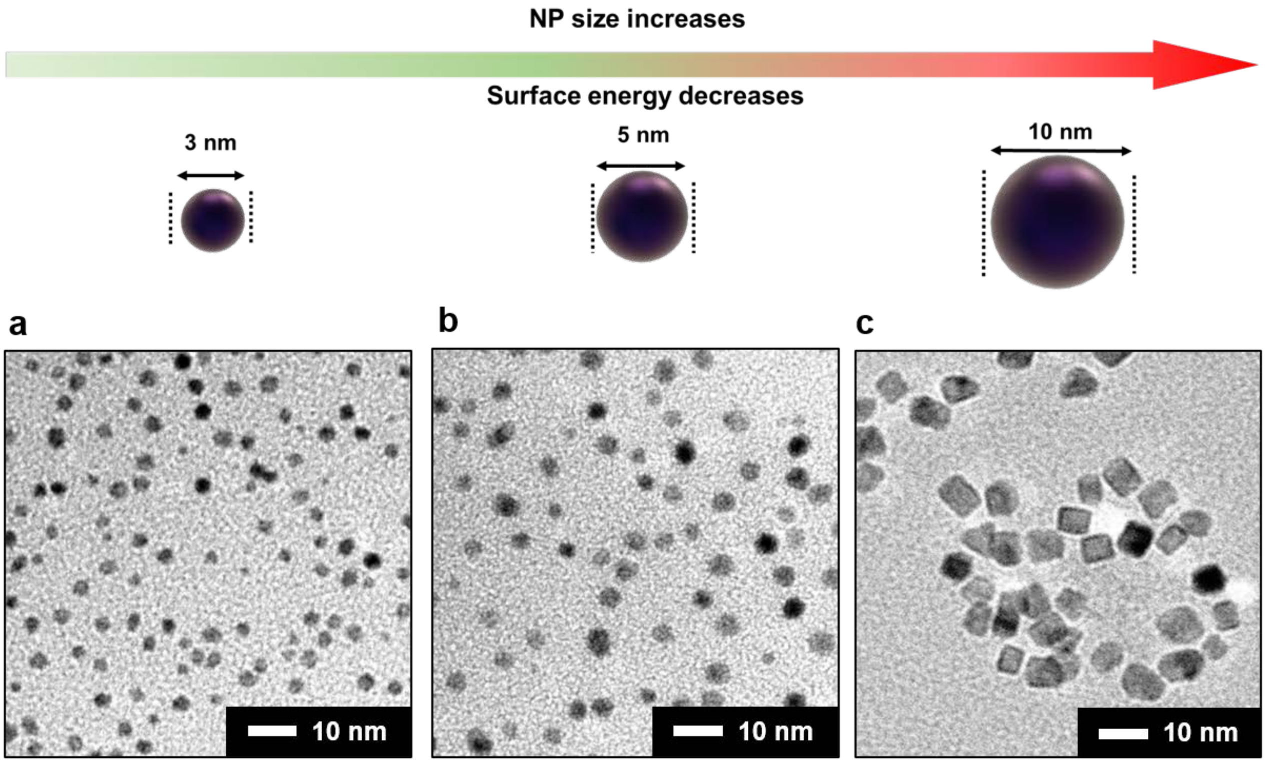

Figure S1. TEM images of a) 3 nm, b) 5 nm and c) 10 nm Pt NPs.

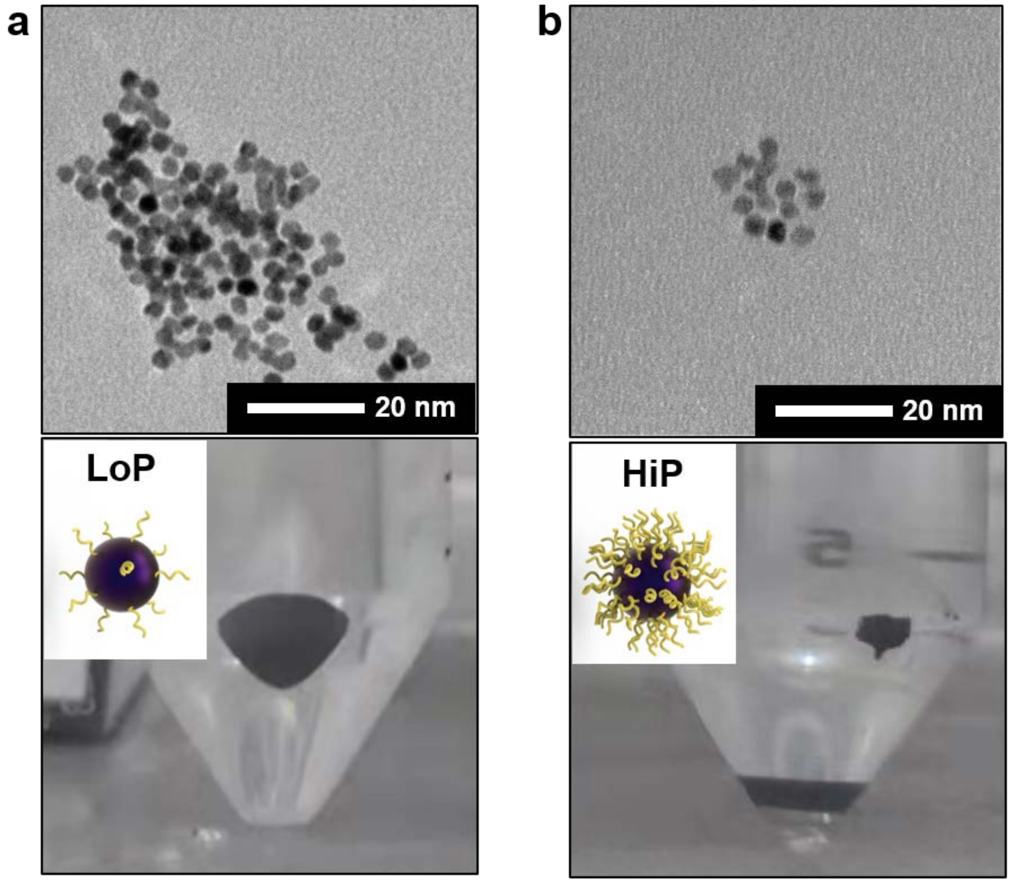

Figure S2. TEM images and soulibility of a) 3 nm LoP Pt-PVP NPs. B) 3 nm HiP Pt-PVP NPs.

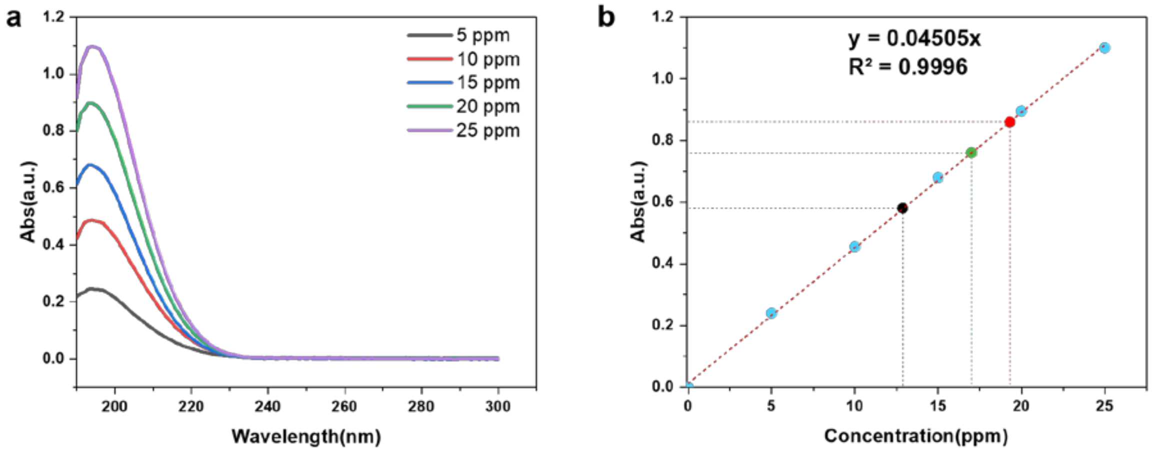

Figure S3. Standard absorption curve of PVP. a) UV-absorption of different concentrations of PVP. b) Calibration curve of UV-absorption at 196 nm and PVP concentrations. The black dot stands for the surface-bound PVP concentration of LoP (12.87 ppm), the green dot stands for the surface-bound PVP concentration of MiP (17 ppm), and the red dot stands for that of HiP (19.30 ppm).

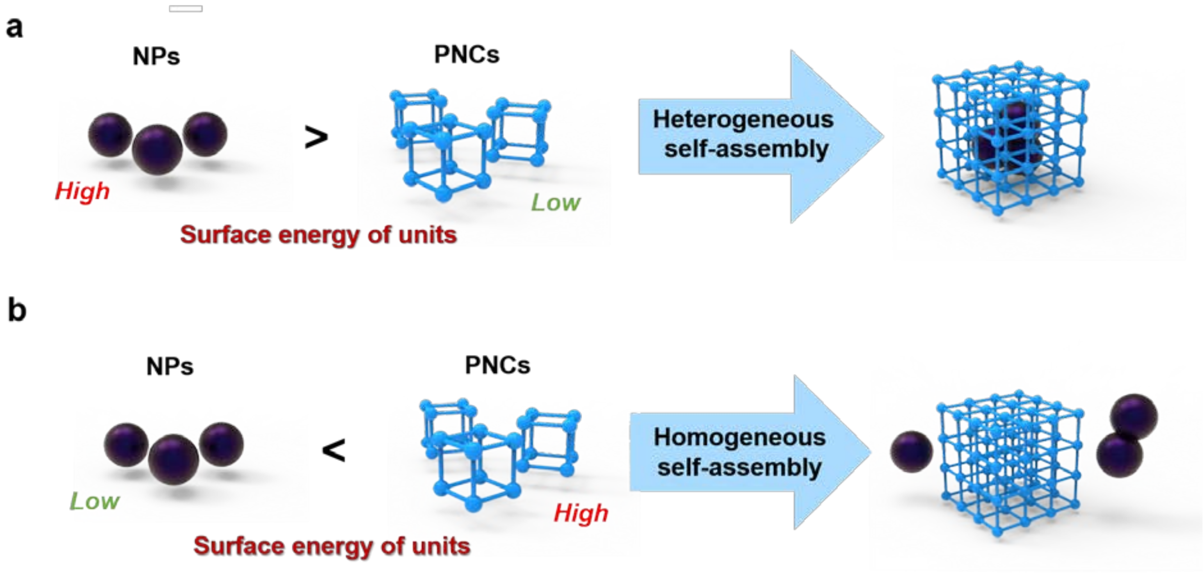

Figure S4. a) The hypothesis of the surface-energy-dominated heterogeneous self-assembly. b) The hypothesis of the surface-energy-dominated homogeneous self-assembly.

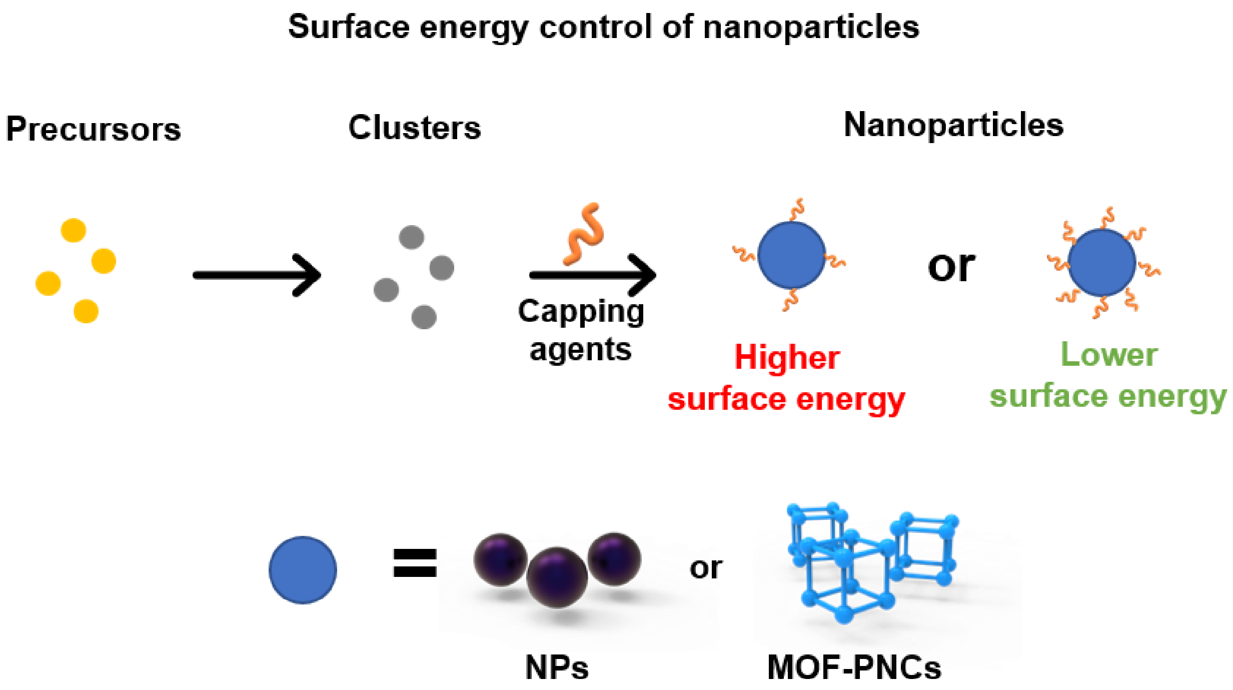

Figure S5. Surface energy control of nanoparticles. Surface energy of both metal NPs and MOF-PNCs can be controlled by capping agents.

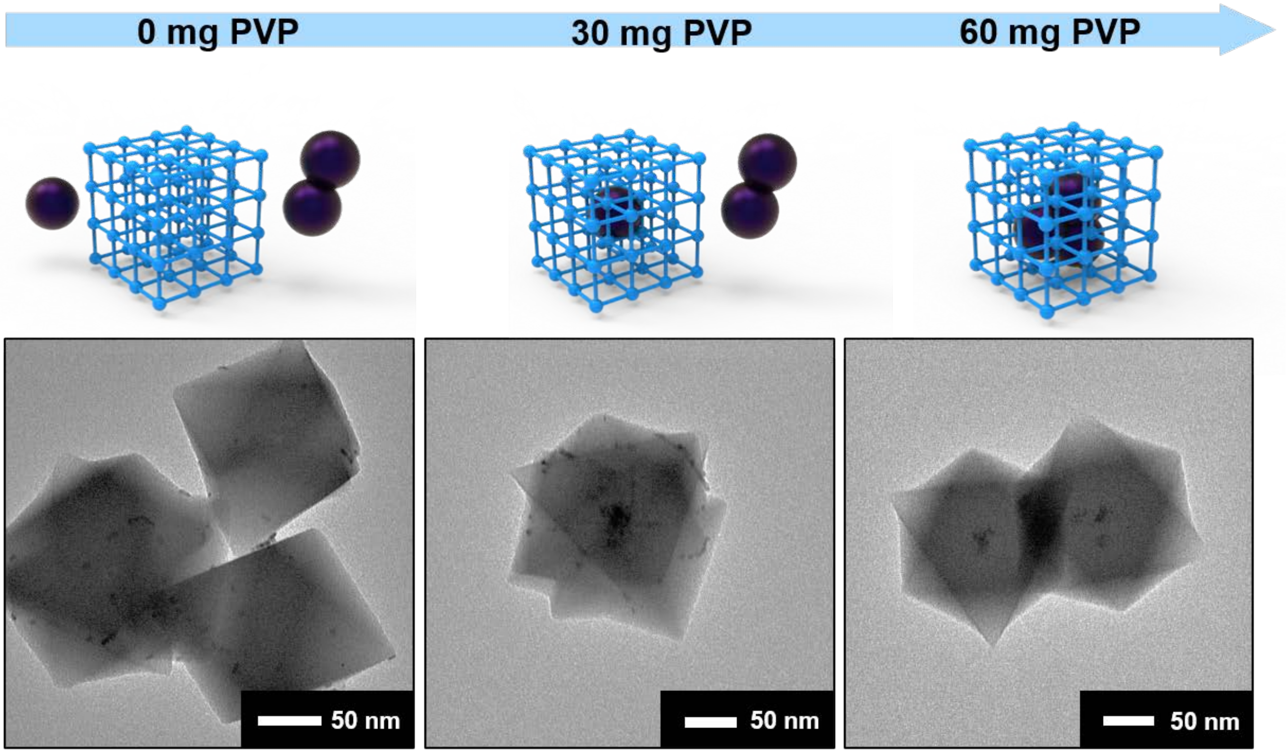

Figure S6. The heterogeneous self-assembly of HiP and UiO-66-PNCs gradually increasing with the increased addition amount of PVP additives.

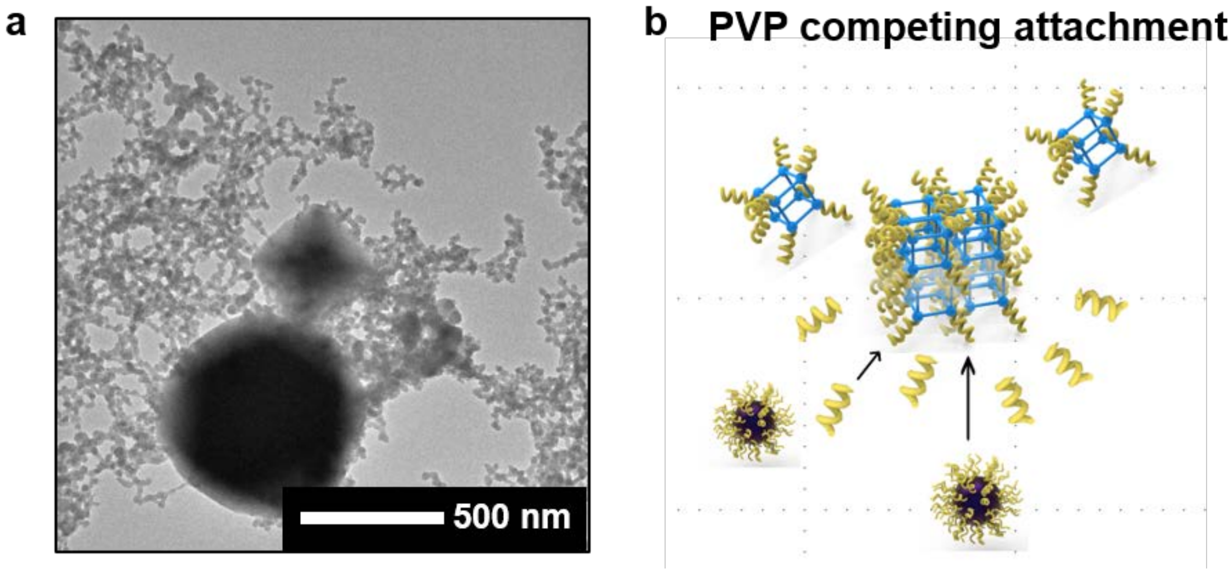

Figure S7. a) TEM image of excessive free PVP additive (500 mg). b) Scheme illustration of competitive attachment of PVP and NPs to MOF-PNCs.

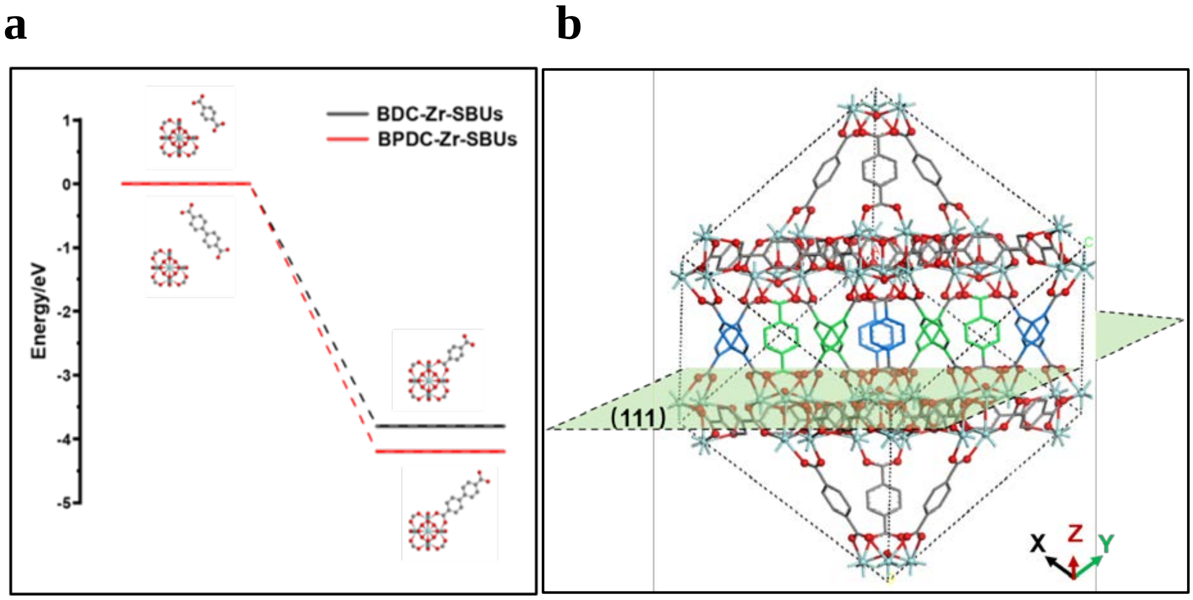

Figure S8. a) The bond-breaking enthalpies of BPDC-Zr-SBUs and BDC-Zr-SBUs from DFT calculations. The calculation detail is in the 1.3. DFT calculation of the Experimental Section. b) Nine ligands need to dissociate from the SBU to create a (111) crystal face in a single unit cell. The green ligands represent a whole one belongs to the unit cell while the blue ligands represent 1/2 of a ligand belongs to the unit cell.

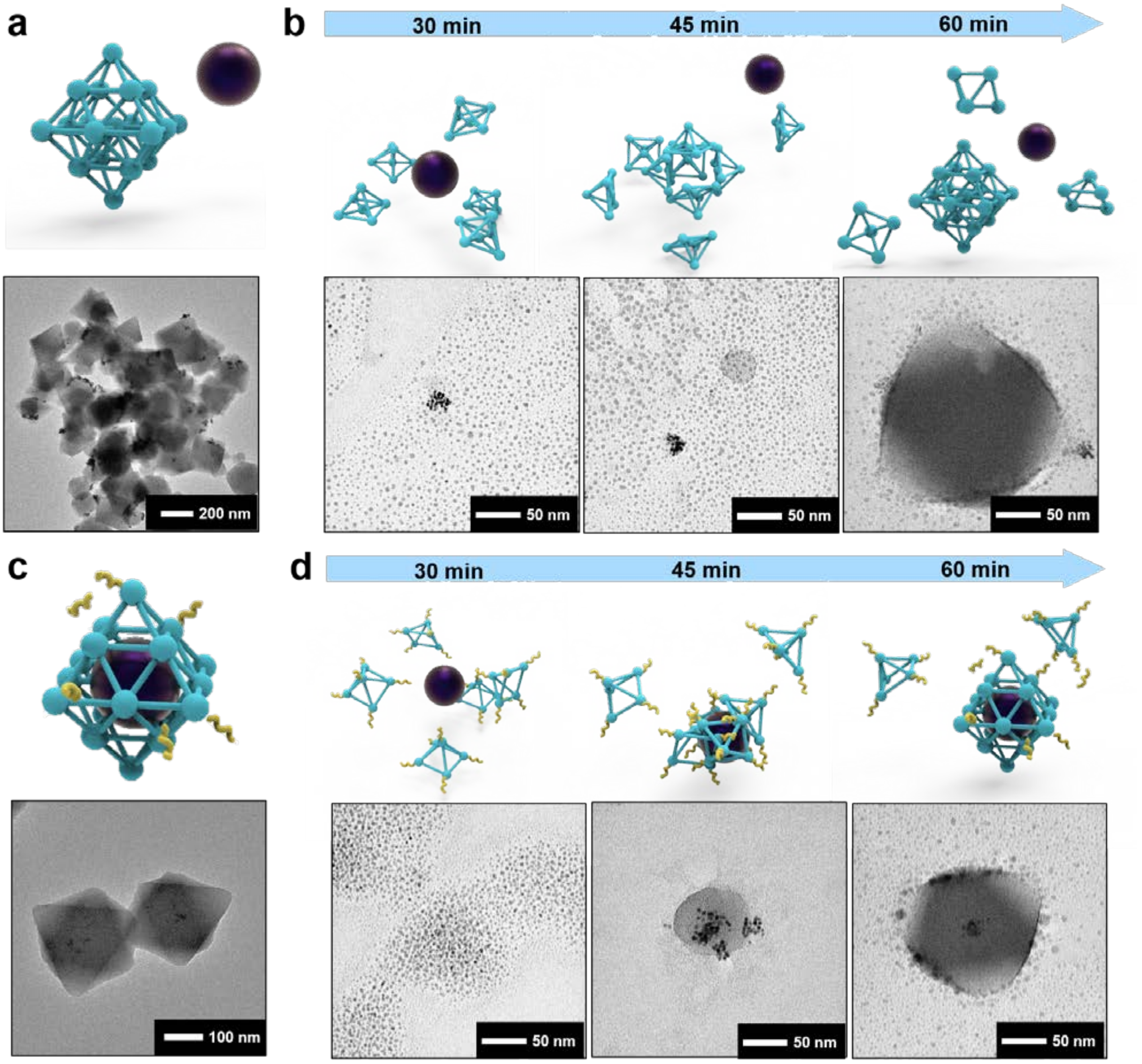

Figure S9. TEM images of a) LoP/UiO-67, b) time-dependent studies of the synthesis stages of LoP/UiO-67, c) LoP@UiO-67 and d) time-dependent studies of the synthesis stages of LoP@UiO-67.

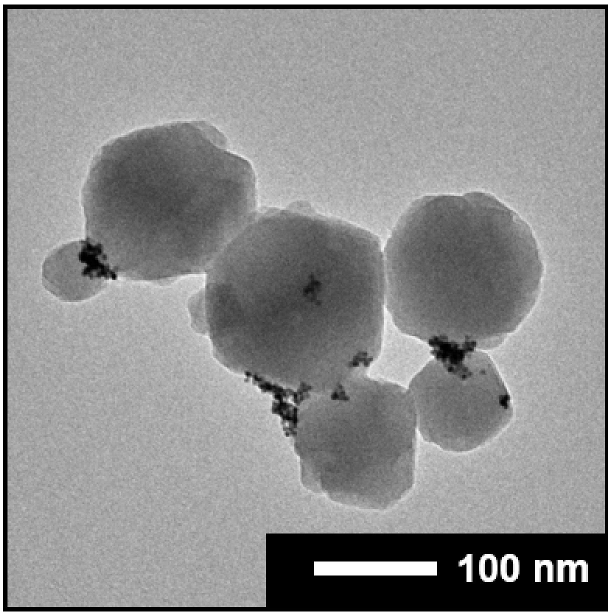

Figure S10. TEM image of HiP/UiO-67.

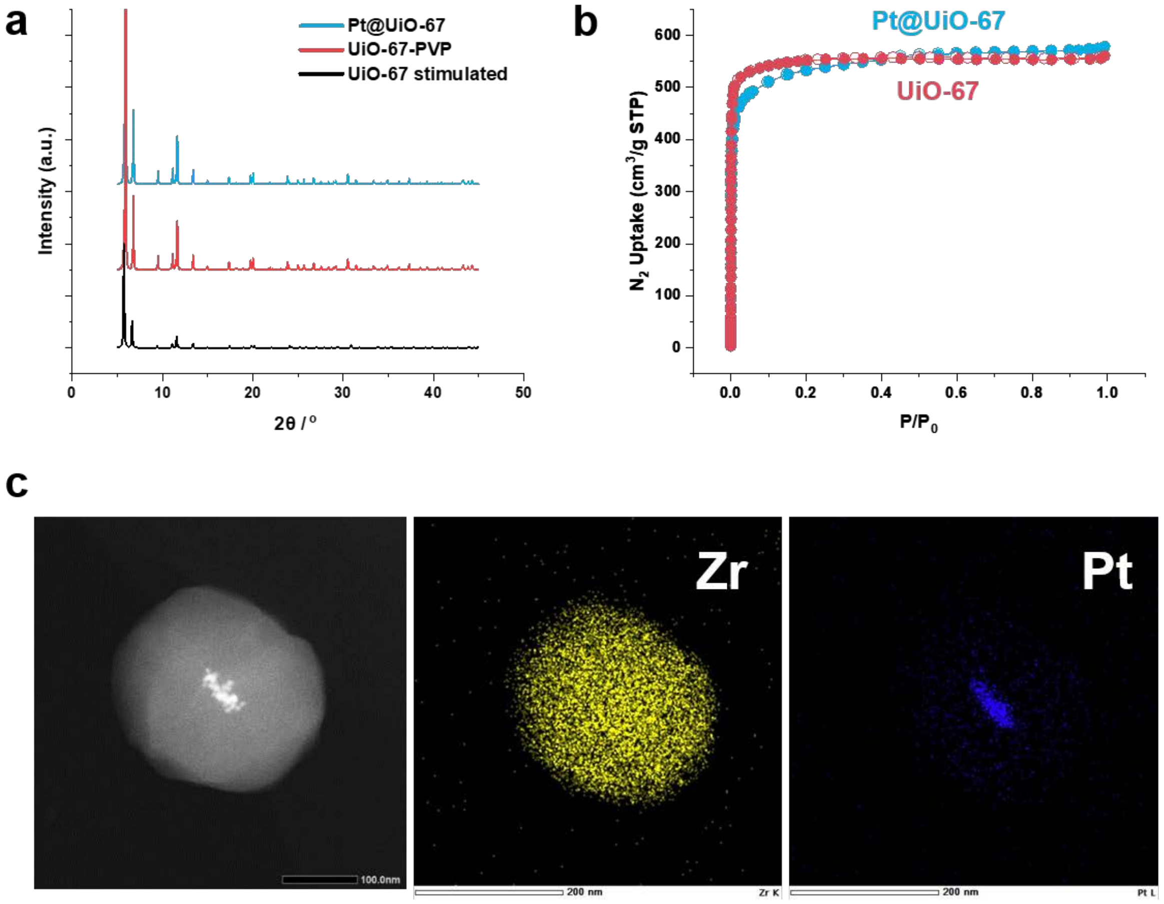

Figure S11. Characterizations of Pt@UiO-67 and UiO-67 by a) PXRD and b) nitrogen adsorption-desorption isotherms. c) STEM-EDS of Pt @UiO-67.

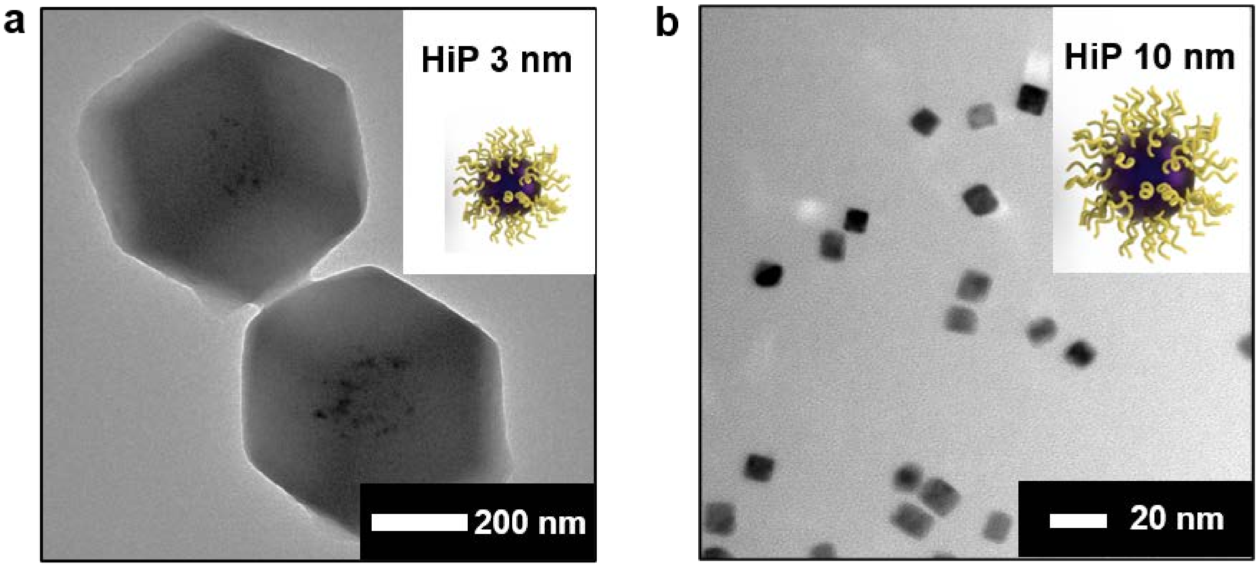

Figure S12. TEM images of a) 3 nm HiP@ZIF-8, b) 10 nm HiP Pt-PVP NPs.

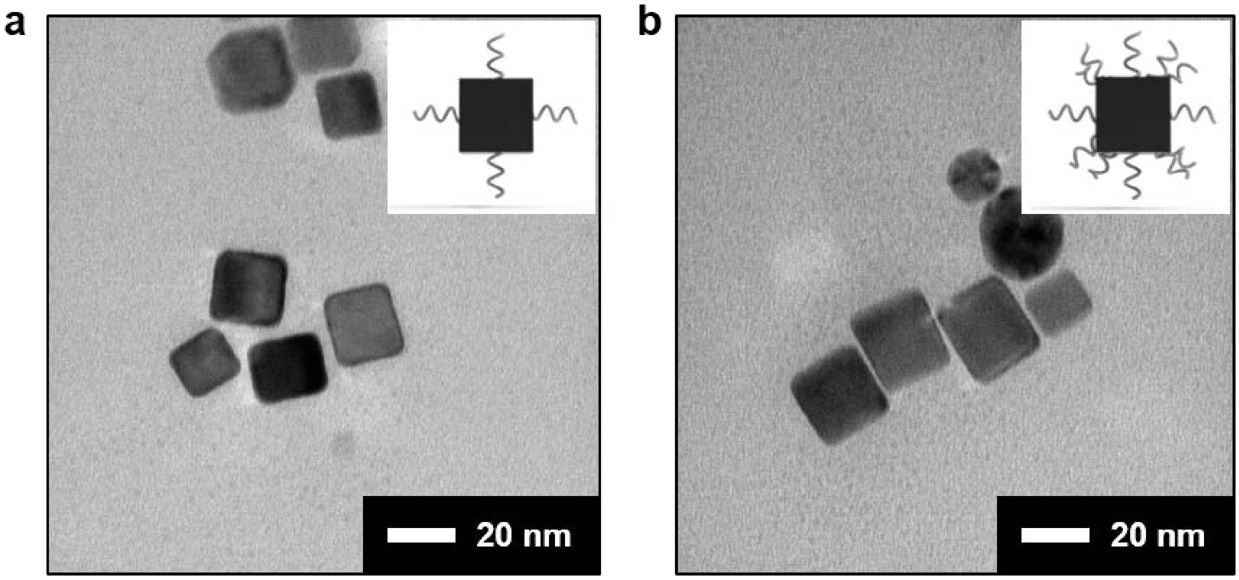

Figure S13. TEM images of a) 20 nm LoC Pd-CTAB NPs, b) 20 nm HiC Pd-CTAB NPs.

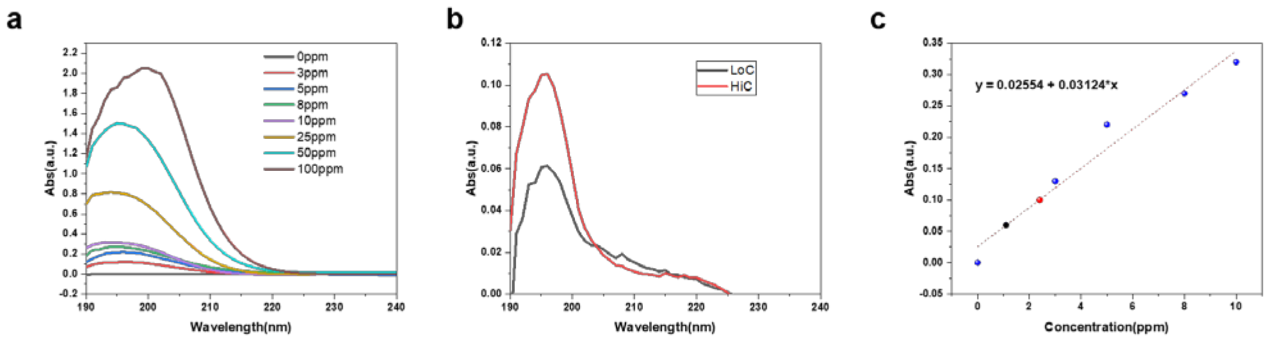

Figure S14. a) Standard absorption curve of CTAB. The UV absorption spectrum of CTAB showed a hyperchromic effect with the increase of concentration, the maximum absorption wavelength and absorbance of CTAB increased with the increase of concentration. [10] b) UV-absorption of the concentrations of LoC (black curve) and HiC (red curve). c) Calibration curve of UV-absorption at 198 nm and CTAB concentrations. The black dot stands for the surface-bound PVP concentration of LoC (1.1 ppm), and the red dot stands for that of HiC (2.4 ppm).

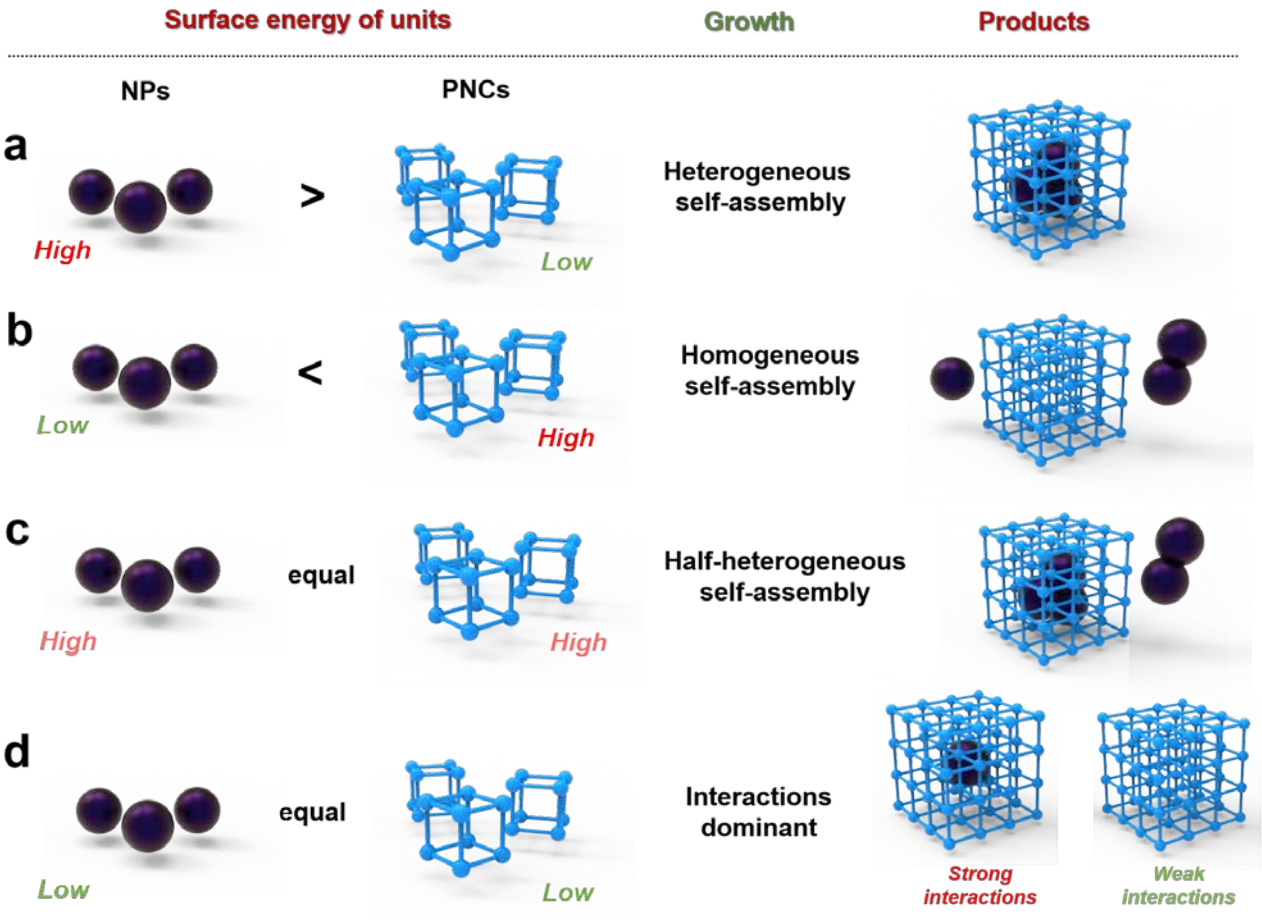

Figure S15. The summary of the formation mechanism of *de novo* NP encapsulation.

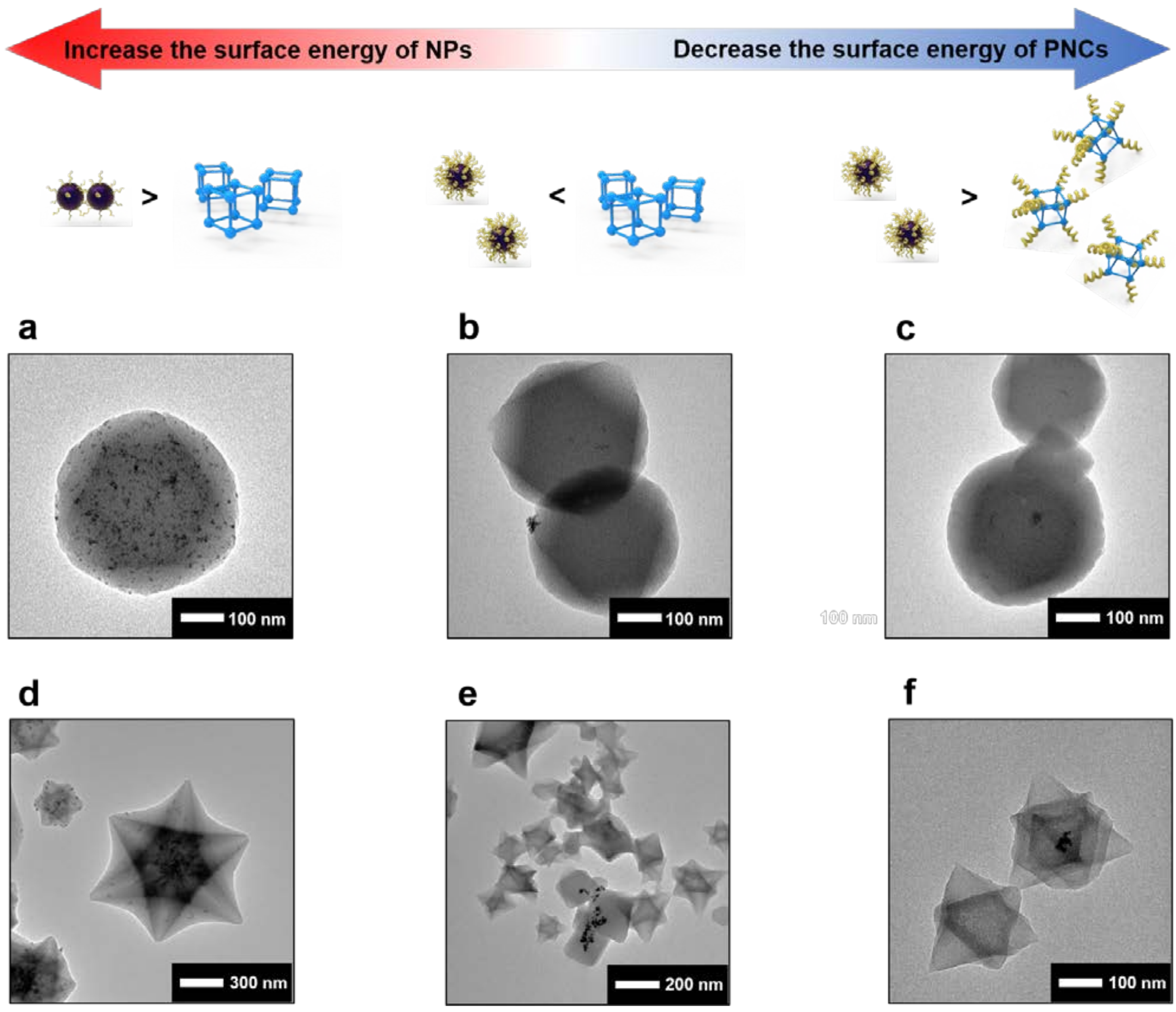

Figure S16. Applications of the synthetic strategy on (a-c) HKUST-1 and (d-f) MIL-101(Fe). a) 3 nm Pt NPs LoP@HKUST-1. b) 10 nm Pt NPs HiP@ZIF-8 without additional free PVP and c) with additional free PVP of 10 mg. d) 3 nm Pt NPs LoP@MIL-101(Fe). e) 10 nm Pt NPs HiP@ MIL-101(Fe) without additional free PVP and f) with additional free PVP of 100 mg.

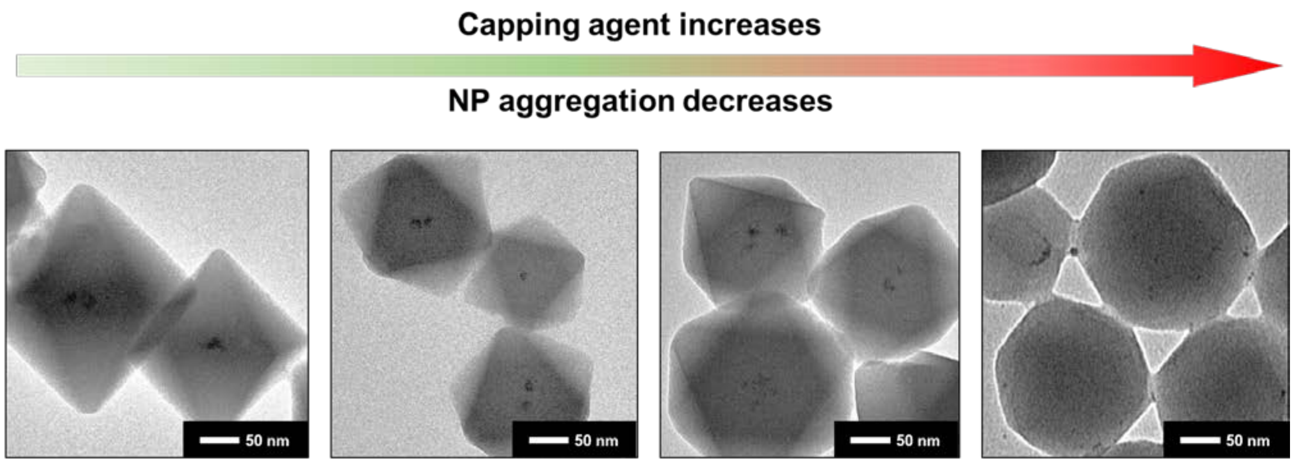

Figure S17. With the increase of additional free PVP from 50 mg, 100 mg, and 200 mg to 500 mg, the dispersity of Pt NPs in UiO-66 gradually increasing, and finally fail encapsulation.

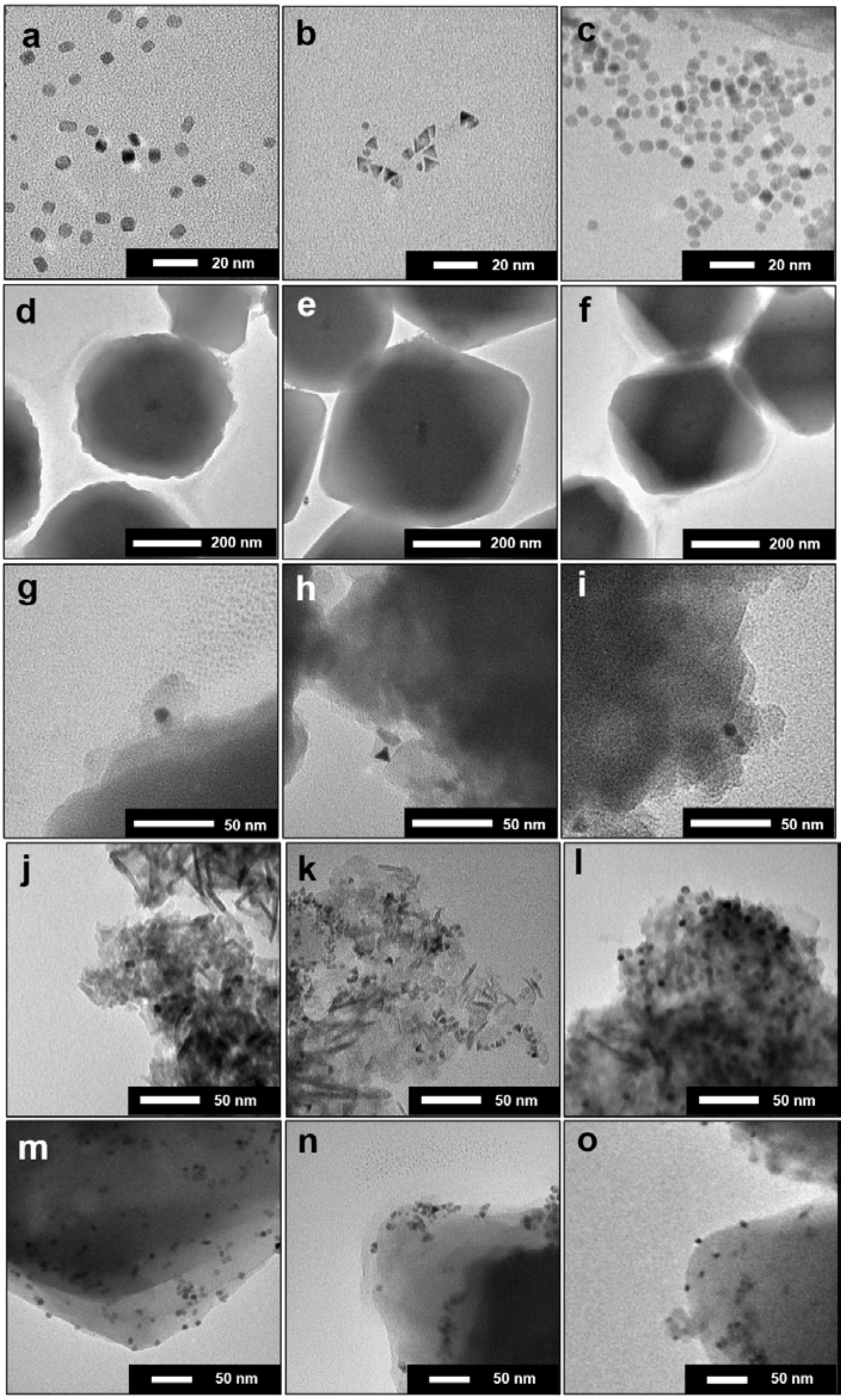

Figure S18. (a-c) The cubic, tetrahedral and cubobtahedral Pd NPs. (d-f) CUB@UiO-67, TET@UiO-67 and COT@UiO-67. (g-i) Edge of small CUB@UiO-67, TET@UiO-67 and COT@UiO-67, the morphologies of Pd NPs are not changed after encapsulation. (j-k) CUB/Al_2O_3, TET/Al_2O_3, COT/Al_2O_3. (m-o) CUB/UiO-67, TET/UiO-67, COT/UiO-67.

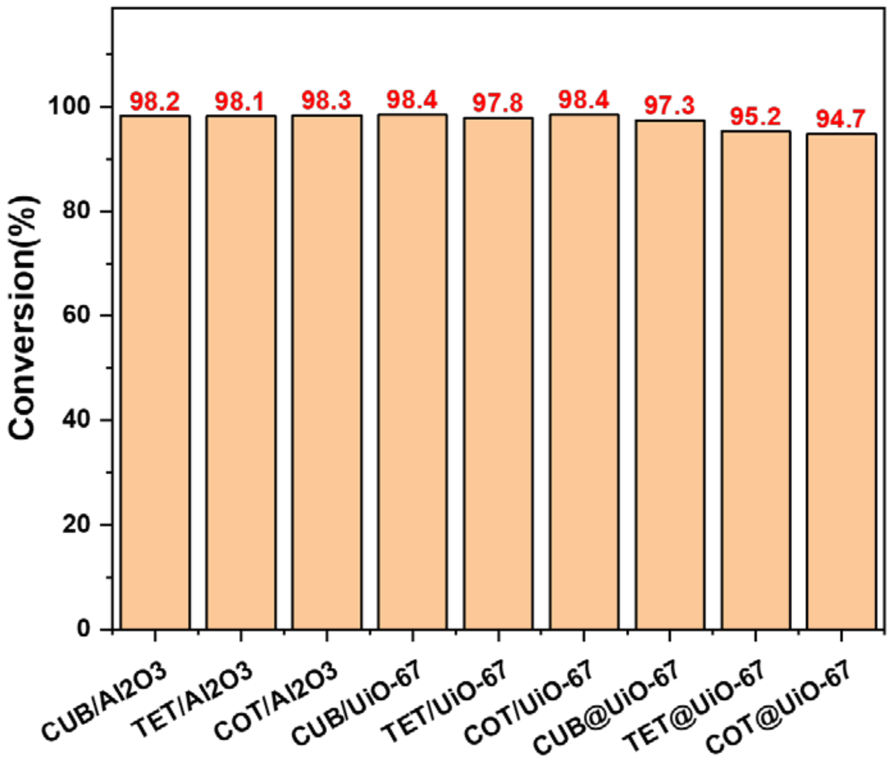

Figure S19. The conversion rate of semihydrogenation of PhA on the as-synthesized samples.

3. Supporting Tables

Table S1. The ratio of Capping agents (CA)/Metal. The concentration of CA is quantified by UV-vis and that of metals are quantified by ICP with the same sample.

Samples	CA	Metal	CA / Metal (ppm)	Ratio
LoP-Pt-3 nm	PVP	Pt	12.87/0.378	34.05
HiP-Pt-3 nm	PVP	Pt	19.30/0.372	51.88
Pt-10 nm	PVP	Pt	7.68/0.213	36.06
HiP-Pt-10 nm	PVP	Pt	9.66/0.197	49.04
CUT-Pd	PVP	Pd	14.68/0.436	33.67
TET-Pd	PVP	Pd	11.68/0.417	28.01
COT-Pd	PVP	Pd	18.98/0.513	37.00
LoC-Pd	CTAB	Pd	1.1/1.236	0.89
HiC-Pd	CTAB	Pd	2.4/0.988	2.43

Table S2. ICP data of Pd amount in CUT@UiO-67, TET@UiO-67 and COT@UiO-67

Samples	Pd (ppm)	Load (%)
CUT@UiO-67	0.112	2.24
TET@UiO-67	0.097	1.94
COT@UiO-67	0.126	2.52

4. References

1 C.-K. Tsung, J. N. Kuhn, W. Huang, C. Aliaga, L.-I. Hung, G. A. Somorjai and P. Yang, *J. Am. Chem. Soc.*, 2009, **131**, 5816–5822.

2 B. T. Sneed, C.-H. Kuo, C. N. Brodsky and C.-K. Tsung, *J. Am. Chem. Soc.*, 2012, **134**, 18417–18426.

3 M. Jin, H. Liu, H. Zhang, Z. Xie, J. Liu and Y. Xia, *Nano Research*., 2011, **4**, 83–91.

4 Y. Wang, S. Xie, J. Liu, J. Park, C. Z. Huang and Y. Xia, *Nano Lett*., 2013, **13**, 5, 2276–2281.

5 W.-S. Lo, L.-Y. Chou, A. P. Young, C. Ren, T. W. Goh, B. P. Williams, Y. Li, S.-Y. Chen, M. N. Ismail, and W. Huang, *Chem. Mater.*, 2021, **33**, 1946–1953.

6 M. J. Katz, Z. J. Brown, Y. J. Colón, P. W. Siu, K. A. Scheidt, R. Q. Snurr, J. T. Hupp and O. K. Farha, *Chem. Commun.*, 2013, **49**, 9449–9451.

7 M. Zhao, K. Yuan, Y. Wang, G. Li, J. Guo, L. Gu, W. Hu, H. Zhao and Z. Tang, *Nature*, 2016, **539**, 76–80.

8 J.-L. Zhuang, D. Ceglarek, S. Pethuraj and A. Terfort, *Adv. Funct. Mater.*, 2011, **21**, 1442–1447.

9 Y. Li, W.-S. Lo, F. Zhang, X. Si, L.-Y. Chou, X.-Y. Liu, B. P. Williams, Y.-H. Li, S.-H. Jung and Y.-S. Hsu, *J. Am. Chem. Soc.*, 2021, **143**, 5182–5190.

10 H.-J. Lu, C. Chen, H.-T. Guo, X.-H. Zhou, J.-F. Dong, X.-L. Hong, X.-F. Li, G.-Y. Zhang, *Acta Chim. Sin.*, 2006, **64**, 2437-2441.
